# Multi-Threshold Image Segmentation of Maize Diseases Based on Elite Comprehensive Particle Swarm Optimization and Otsu

**DOI:** 10.3389/fpls.2021.789911

**Published:** 2021-12-13

**Authors:** Chengcheng Chen, Xianchang Wang, Ali Asghar Heidari, Helong Yu, Huiling Chen

**Affiliations:** ^1^College of Computer Science and Technology, Jilin University, Changchun, China; ^2^Key Laboratory of Symbolic Computation and Knowledge Engineering of Ministry of Education, Changchun, China; ^3^Chengdu Kestrel Artificial Intelligence Institute, Chengdu, China; ^4^School of Surveying and Geospatial Engineering, College of Engineering, University of Tehran, Tehran, Iran; ^5^College of Information Technology, Jilin Agricultural University, Changchun, China; ^6^College of Computer Science and Artificial Intelligence, Wenzhou University, Wenzhou, China

**Keywords:** non-local mean filtering, enhanced comprehensive learning particle optimizer, Otsu, multi-threshold image segmentation, maize disease image

## Abstract

Maize is a major global food crop and as one of the most productive grain crops, it can be eaten; it is also a good feed for the development of animal husbandry and essential raw material for light industry, chemical industry, medicine, and health. Diseases are the main factor limiting the high and stable yield of maize. Scientific and practical identification is a vital link to reduce the damage of diseases and accurate segmentation of disease spots is one of the fundamental techniques for disease identification. However, one single method cannot achieve a good segmentation effect to meet the diversity and complexity of disease spots. In order to solve the shortcomings of noise interference and oversegmentation in the Otsu segmentation method, a non-local mean filtered two-dimensional histogram was used to remove the noise in disease images and a new elite strategy improved comprehensive particle swarm optimization (PSO) method was used to find the optimal segmentation threshold of the objective function in this study. The experimental results of segmenting three kinds of maize foliar disease images show that the segmentation effect of this method is better than other similar algorithms and it has better convergence and stability.

## Highlights

- The Otsu is combined with an elite comprehensive particle swarm algorithm for image segmentation.
- A non-local mean filtered 2D histogram is combined to remove noise.
- The GCLPSO is the first time applied to the segmentation of a variety of diseases of maize.
- The GCLPSO performs better than other similar algorithms under multi-threshold segmentation.

## Introduction

Diseases often occur during maize cultivation and failure to prevent and control them in time can decrease corn yield and quality, making a loss of economic benefits for the grower. According to the data reported by the Food and Agriculture Organization (FAO) of the United Nations, the annual natural loss rate caused by agricultural pests and diseases is more than 37% and agricultural pest and disease identification and control are of great importance to improve agricultural production (Dhami et al., 2015). Traditional manual identification methods rely on experience, high labor costs, subjective factors, and lack of accuracy (Gao and Lin, 2019). In recent years, computer vision and image processing methods have gradually developed; the method is more objective and supports the real-time online diagnosis, bringing new opportunities to solve agricultural disease diagnosis, reduce economic losses, and improve crop yields (Wang et al., 2019).

Diseases are the main causal factors affecting crop growth and in order to improve the quality and yield of crops, targeted screening and diagnosis are needed during the growth process. Crop disease recognition technology based on machine vision image processing usually includes disease image preprocessing, image segmentation, feature extraction, image recognition, and interpretation (Iqbal et al., 2018). The higher the accuracy of image segmentation, the higher the accuracy of feature extraction and image recognition. Therefore, higher accuracy segmentation methods are the main research direction for scholars in disease identification and diagnosis. The mainstream agricultural disease image segmentation techniques are mainly based on clustering methods (Wang et al., 2018), region growth methods (Jothiaruna and Sundar, 2020), edge detection methods (Shaikh et al., 2017), threshold segmentation methods (Wang et al., 2013), heuristic algorithm methods (Zhou et al., 2018), machine learning, and deep learning methods (Ale et al., 2019). Although there are many novel and effective image segmentation methods in recent literature (Elaziz et al., 2020; Rodriguez-Esparza et al., 2020; Zhao et al., 2020a,b, 2021), there is not yet a general robustness well-adapted segmentation method that can be applied to multiple scenes due to the interference of many crop species, complex background information, diverse and disorderly disease spot morphological texture colors, multiple disease spot interference, blurred leaf surface texture, and disease spot boundaries. The currently available segmentation methods and their advantages and disadvantages are shown in following Table 1.

**Table 1:** Existing segmentation methods and their advantages and disadvantages.

**Segmentation method**	**Advantages**	**Disadvantages**
Edge detection	Fast search detection and good detection of edges	Cannot get a better regional structure; The conflict between noise immunity and detection accuracy during edge detection
Region growth	Effectively overcome the disadvantage of small continuous image segmentation space existing; Better regional characteristics	Easy to cause excessive segmentation of images, complex and computationally intensive
Clustering	High sensitivity to initial settings, and sometimes it needs human decisions within initialization	No consideration of spatial information, sensitive to noise and gray scale inhomogeneity
Threshold	Direct use of the grayscale characteristics of the image, so the calculation is simple, efficient, and fast	Sensitive to noise, not obvious to grayscale differences and different target grayscale values have overlapping segmentation is not obvious, need to find a suitable threshold with other methods
Mathematical morphology	Good positioning effect, high segmentation accuracy, good anti-noise performance	High requirement for accuracy of pre-processed images; otherwise, the speed of calculation is reduced
Deep learning	Resolve noise and unevenness in images	Requires a large amount of data, very slow, complex structure, segmentation accuracy is related to the amount of data

Threshold-based segmentation methods are computationally efficient, straightforward, and widely used in multiple fields and crop image recognition. Subramani et al. (2019) presented a method that combines non-local median filter and double line clustering to analyze the anthracnose, blight disease in grapes, tomato, and cucumber. Xiong et al. (2020) proposed an automatic image segmentation algorithm (AISA) based on the GrabCut algorithm that automatically removes the background information of the images while retaining the disease spots. Kumari et al. (2019) presented a novel approach based on the simple linear iterative clustering segmentation method to detect disease in plant leaves. Yan et al. (2018) proposed that extract the H channel information in the HSI, more common, components hue (H), saturation (S), brightness(I) and use each pixel and its local average value to form a two-dimensional (2D) histogram, then segment color space the image by the optimal threshold of the Otsu algorithm in the polar diameter information to improve the segmentation accuracy of rice blast images. Zhang et al. (2019) presented a novel hybrid clustering segmentation method of plant disease leaf image. Hu et al. (2017) proposed an improved Chan–Vese (C-V) model for wheat leaf lesion segmentation. Gao and Lin (2019) proposed a fully automatic segmentation method using leaf images of medicinal plants in complex backgrounds with vein enhancement and extraction in the images. Among the threshold image segmentation methods, the Otsu (Merzban and Elbayoumi, 2019) segmentation method is one of the classical threshold segmentation methods, which has obvious disadvantages of misclassification and computational complexity using grayscale histograms and cannot be well-adapted to complex and diverse crop disease images. Mittal and Saraswat (2018) proposed a 2D histogram with non-local mean filtering, which can effectively reduce the loss of image details. However, the Otsu method finds the optimal threshold using the exhaustive method, the complexity grows exponentially with the increase of the number of thresholds, and there are obvious shortcomings in the computational performance. The application of swarm intelligence algorithm is a bionic approach to solve optimization problems with intelligence, parallelism, and robustness, which is widely used in thresholding optimization problems. Jia et al. (2019a) used improved moth flame optimization (MFO) for multistage thresholding segmentation of color images. Jia et al. (2019b) proposed an improved multilevel optimization algorithm based on Lévy flight for multi-threshold color image segmentation. Kotte et al. (2018) proposed a fast multi-thresholding method for gray image segmentation based on a differential evolution algorithm. Bao et al. (2019) proposed a novel hybrid Harris Hawks optimization method for multilevel threshold segmentation of color images.

**Figure 1:**
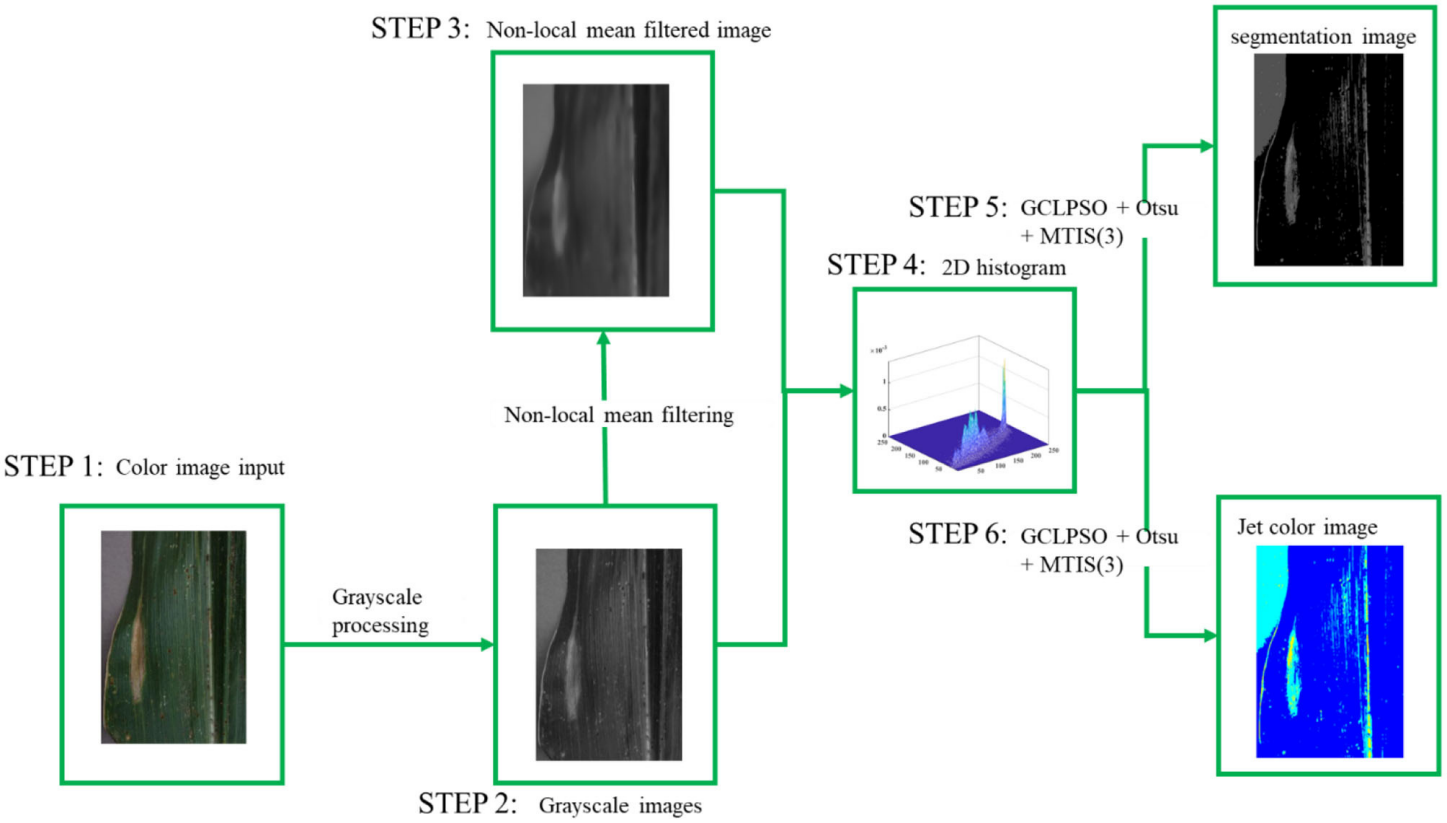
Flowchart of multi-threshold image segmentation of maize diseases using GCLPSO + Otsu.

In this study, non-local mean filtering of 2D histogram Otsu was used to segmentation for multi-threshold image processing and enhanced comprehensive learning particle swarm optimizer with elite-based dominance scheme (GCLPSO) (Chen et al., 2020) was used to find the optimal threshold, which was used to achieve higher convergence speed and accuracy, can quickly achieve convergence to the optimal value, and improve the efficiency of image segmentation. The methods were applied to maize leaf disease images in the Plant Village^1^ public database. The experimental results indicated that the method effectively improved the segmentation of the three maize disease spot images and could obtain more apparent disease spot areas. To verify the experimental validity, the used GCLPSO was compared with the original CLPSO (Liang et al., 2006) and two of improved algorithms such as Sine Cosine Algorithm and Differential Evolution (SCADE) (Nenavath and Jatoth, 2018), modified sine cosine algorithm (m_SCA) (Qu et al., 2018) and three original algorithms such as salp swarm algorithm (SSA) (Aljarah et al., 2018; Faris et al., 2018; Abbassi et al., 2019), SCA (Oliva et al., 2018; Qu et al., 2018; Kuo et al., 2020), and Slime mould algorithm (SMA) (Abbassi et al., 2019), respectively. The segmentation experiments with multiple thresholds were also performed separately. In addition, the feature similarity (FSIM) index (Zhang et al., 2011), peak signal-to-noise ratio (PSNR) (Setiadi, 2020), and structural similarity (SSIM) index (Wang et al., 2004) were used to compare the image segmentation results for evaluation and the mean, variance, and the Wilcoxon signed-rank (Garcia et al., 2010) tests were used to analyze the evaluation results. Through a series of analyses and comparisons of experimental results, GCLPSO for non-local mean filtering of 2D histogram Otsu multi-threshold image processing outperforms other algorithms in terms of overall performance and can effectively segment corn leaf disease images. Figure 1 shows the steps of image segmentation based on this method.

The rest of this study is organized as follows. Chapter 2 introduces the multi-threshold Otsu segmentation. Chapter 3 introduces the non-local mean filtered 2D histogram. Chapter 4 introduces GCLPSO. Chapter 5 conducts a series of comparison experiments between GCLPSO and other optimal thresholding methods, and chapter 6 summarizes the whole paper and the direction of future work.

## Multi-Threshold OTSU Segmentation

Crop disease image segmentation is mainly concerned with separating the disease spots of a crop from the leaves or other backgrounds containing the leaves. Multi-threshold segmentation is an integral part of digital agricultural image processing. It mainly refers to marking out the targets of interest in an image by setting multiple thresholds. The selection of thresholds is critical and related to the good or bad results after segmentation. Otsu method is a more common and perfect method in multi-threshold image segmentation (MTIS), which was proposed by Japanese scholar Otsu in 1979 and it is also called the maximum interclass variance method and its principle is that the interclass variance between foreground and background images is maximum after image binarization segmentation according to the thresholds obtained by the Otsu method (Merzban and Elbayoumi, 2019).

For an *M* × *N* image *I, x* is the row coordinates of image pixel points, *y* is the column coordinates of image pixel points, where 0 ≤ *x* < *m*, 0 ≤ *y* < *n*. The image gray level is *S* = {0, 1, …, *L* − 1}, (*L* = 256) and the number of all the image pixel points is denoted as: *m* × *n*. *G* is the corresponding averaged image; then pixel gray level in *G* can be defined as follows:

(1) $$\begin{array}{r} G\left(x,\;y\right)=\overset{x+\frac{k-1}{2}}{\underset{\tilde{x}=x-\left(k-\frac{1}{2}\right)}{\sum }}\overset{y+\frac{k-1}{2}}{\underset{ỹ=x-\left(k-\frac{1}{2}\right)}{\sum }}I\left(\tilde{x},ỹ\right) \end{array}$$

where *I*(*x, y*) and *G*(*x, y*) represent the gray level of the pixel at *x, y* in *I* and *G*, respectively. *k* represents the size of the filter and the value of *k* is set to be 3 in this study. Let *i, j* be pixel gray level of original image and averaged image; then *i, j* is a gray level pair representing that the pixel gray level in image *I* is *i* and the gray level of the corresponding pixel at the same location in the averaged image *G* is *j*. Suppose *f*_*ij*_ is the pixel number of *i, j*, then the 2D probability function can be defined as:

(2) $$\begin{array}{r} P_{ij}=\frac{f_{ij}}{m\times n} \end{array}$$

where *i,j*ϵ (0, *L* – 1) and $\sum_{i}\sum_{j}P_{ij}=1$. The average vector of the 2D histogram is as follows:

(3) $$\begin{array}{r} \mu_{T}={\left(\mu_{Ti},\mu_{Tj}\right)}^{T}={\left(\overset{L-1}{\underset{i=0}{\sum }}\overset{L-1}{\underset{j=0}{\sum }}iP_{ij},\overset{L-1}{\underset{i=0}{\sum }}\overset{L-1}{\underset{j=0}{\sum }}jP_{ij}\right)}^{T} \end{array}$$

A given threshold pair (s, t), pixels can be partitioned into two sets, *C*_0_ and *C*_1_ (background and foreground) and the class occurrence probabilities can be expressed as:

(4) $$\begin{array}{r} \omega_{0\;}=P\left(C_{0}\right)=\overset{s}{\underset{i=0}{\sum }}\overset{t}{\underset{j=\;0}{\sum }}P_{ij},\\ \omega_{1}=P\left(C_{1}\right)=\overset{L-1}{\underset{i=s+1}{\sum }}\overset{L-1}{\underset{j=t+1}{\sum }}P_{ij}. \end{array}$$

The corresponding mean vectors of *C*_0_ and *C*_1_ are:

(5) $$\begin{array}{r} \mu_{0}=\;{\left(\mu_{0i},\mu_{0j}\right)}^{T}={\left(\overset{s}{\underset{i=0}{\sum }}\overset{t}{\underset{j=0}{\sum }}\frac{iP_{ij\;}}{\omega_{0\;}},\overset{s}{\underset{i=0}{\sum }}\overset{t}{\underset{j=0}{\sum }}\frac{jP_{ij\;}}{\omega_{0\;}}\right)}^{T},\\ \mu_{1}=\;{\left(\mu_{1i},\mu_{1j}\right)}^{T}={\left(\overset{L-1}{\underset{i=s+1}{\sum }}\overset{L-1}{\underset{j=t+1}{\sum }}\frac{iP_{ij\;}}{\omega_{1\;}},\overset{L-1}{\underset{i=s+1}{\sum }}\overset{L-1}{\underset{j=t+1}{\sum }}\frac{iP_{ij\;}}{\omega_{1\;}}\right)}^{T}. \end{array}$$

If we ignore the diagonal data far away from the 2D histogram in the image, we can get the following formula can be obtained:

(6) $$\begin{array}{r} \omega_{0}+\omega_{1}≅1,\;\mu_{T}={\omega_{0}\mu }_{0}+{\omega_{1}\mu }_{1} \end{array}$$

The expression between classes in the 2D Otsu algorithm is as follows:

(7) $$\begin{array}{r} tr\left(\sigma_{B}\right)=tr\left(\overset{1}{\underset{K=0}{\sum }}\omega_{K}\left[{\left(\mu_{K}-\mu_{T}\right)\left(\mu_{K}-\mu_{T}\right)}^{T}\right]\right) \end{array}$$

Then, we calculate the maximum value in Equation (8) to get the best threshold pair (s^*^, t^*^):

(8) $$\begin{array}{r} \left(s^{*},t^{*}\right)=argmax_{0<s<L,0<t<L}\left[tr\left(\sigma_{B}\right)\right] \end{array}$$

The larger the interclass variance, the closer the threshold to the correct image segmentation. The essence of threshold segmentation can be seen as an optimization problem of classifying image pixels according to multiple pixel gray levels, translating into a mathematical model problem of solving the objective function with the best quality. The Otsu thresholding segmentation uses an iterative approach to find the threshold that maximizes the between-class variance for the final desired threshold, so the complexity of algorithm grows exponentially as the number of thresholds and dimensions increases. In agricultural disease diagnosis applications, it could not meet the requirements of rapid diagnosis. Swarm intelligence optimization algorithm can quickly improve computational efficiency; many scholars at home and abroad carry out much evolutionary research.

## Non-local Mean Filtered 2D Histogram

In reality, the occurrence of crop diseases is not a single event, but may be accompanied by damage caused by insects, multiple diseases or weather, and other disasters; in the image capture process, the different angles of shooting disease spots, sunlight, and room light cause uncertainty for segmentation; the irregular diversity of disease spots themselves is also the main noise that causes the inability to extract disease spots accurately. The noise can bring great difficulties to solve the threshold processing. A common approach that requires noise reduction at the source to enhance performance is to smooth the image in priority. Non-local means filtering (NL-means) is a novel denoising technique proposed in recent years. This method makes full use of the redundant information in the image and can maintain the maximum detail features of the image while denoising. A brief algorithm with superior performance characterizes the method. The basic idea is that the estimate of the current pixel is obtained by a weighted average of pixels in the image that has a similar neighborhood structure to it. It can effectively remove most of the noise on crop leaves for other reasons and is an effective method for removing crop disease image noise.

In image *I*, the grayscale values of *p* and *q* corresponding pixels are *I*(*p*) and *I*(*q*), respectively, then the non-local mean value of the image is calculated as follows:

(9) $$\begin{array}{r} O\left(p\right)=\frac{\sum_{q\in I}I\left(q\right)\omega \left(p,q\right)}{\sum_{q\in I}\omega \left(p,q\right)} \end{array}$$

(10) $$\begin{array}{r} \omega \left(p,q\right)=\exp^{-\frac{|\mu \left(p\right)-\mu \left(q\right){|}^{2}}{\sigma^{2}}} \end{array}$$

(11) $$\begin{array}{r} \mu \left(p\right)=\frac{1}{m\times m}\underset{i\in L\left(p\right)}{\sum }I\left(i\right) \end{array}$$

(12) $$\begin{array}{r} \mu \left(q\right)=\frac{1}{m\times m}\underset{i\in L\left(q\right)}{\sum }I\left(i\right) \end{array}$$

*O*(*p*) is the non-local mean filtered value of pixel *p*, ω(*p, q*) is the weight of *p* pixels and *q* pixels, σ is the SD, μ(*p*) and μ(*q*) are the local means of *p* and *q*, *L*(*p*) is the *m* × *m* domain window around *p* pixels, and *L*(*q*) is the *m* × *m* domain window around *q* pixels. *I*(*x, y*) is the grayscale value, *g*(*x, y*) is the non-local mean filtered values, then *i* in the new histogram horizontal and vertical coordinates (*i, j*) denotes the grayscale values, and *j* denotes the non-local mean filtered value. Meanwhile, the size of the original image and the size of the generated non-local mean filtered image are kept the same; therefore, the corresponding non-local mean filtered 2D histogram can be generated from the non-local mean image and the grayscale image. Furthermore, by normalization process, the following equation shows:

(13) $$\begin{array}{r} P_{ij}=\frac{h\left(i,j\right)}{M\times N} \end{array}$$

**Figure 2:**
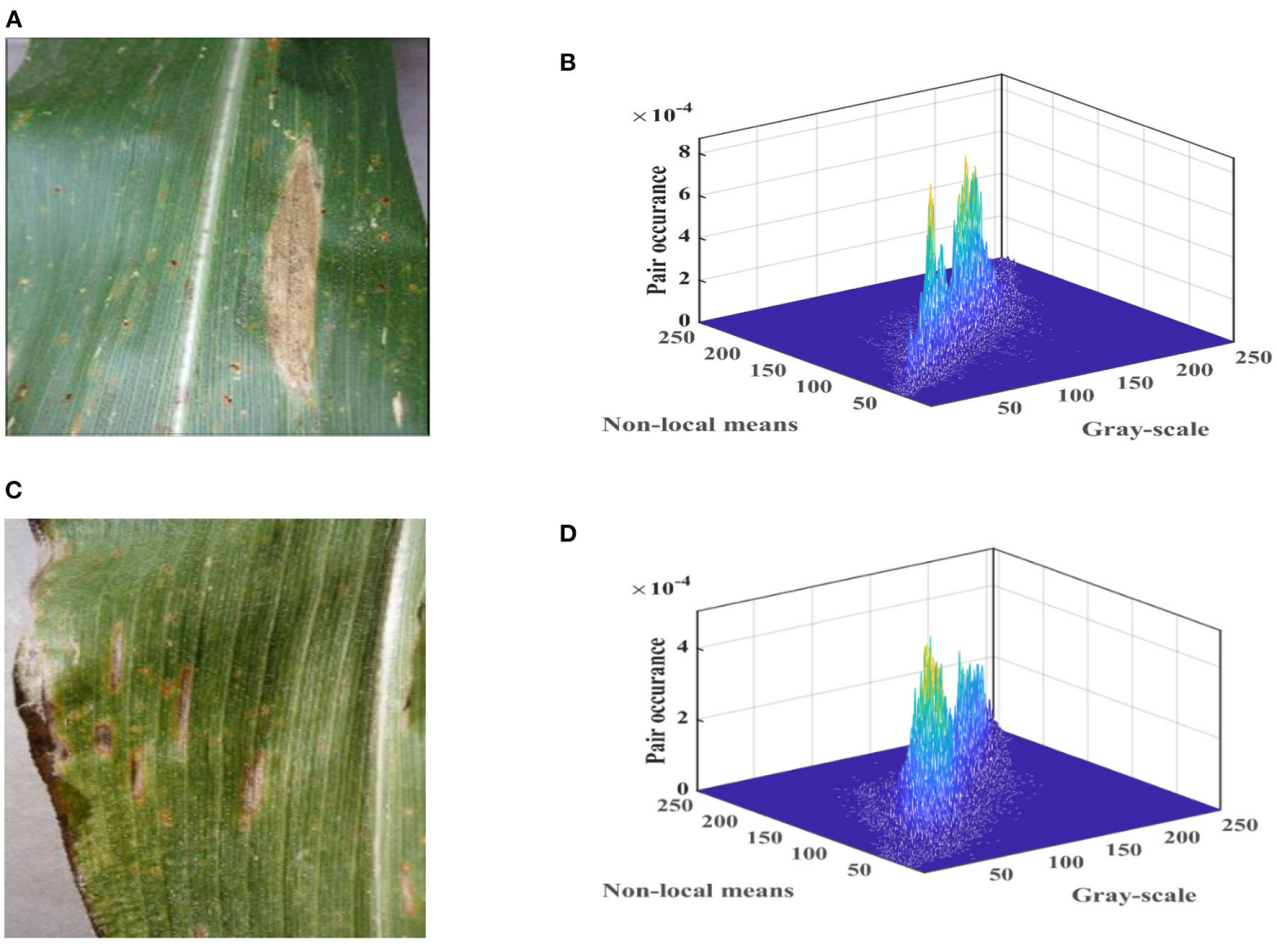
Color images and three-dimensional (3D) views of two-dimensional (2D) histograms for maize leaf spot disease and maize gray spot disease in the Plant Village.

The final 2D histogram can be formed. Figure 2 shows the three-dimensional (3D) views of 2D histograms formed by normalizing it with Equation (11).

## Enhanced Comprehensive Learning Particle Swarm Optimizer

There have been many studies on swarm intelligence optimization methods for finding optimal thresholds of images. In this study, we refer to a recently improved comprehensive particle swarm algorithm, GCLPSO, which improves the exploration and detection capability of CLPSO and improves the ability to find the optimal threshold and this study presents this algorithm.

### Comprehensive Learning Particle Swarm Optimizer

The CLPSO algorithm was proposed by Liang et al. (2006). It uses a new comprehensive learning strategy (CLS) to update the velocity of particles using the personal best position *pbest* of other particles. CLS can maintain the diversity of the population and prevent premature maturation. The velocity and position update formula in the CLPSO algorithm is shown as follows:

(14) $$\begin{array}{r} v_{id}=w*v_{id}+c*r_{id}\left(pbest_{f_{i}\left(d\right),d}-x_{id}\right) \end{array}$$

(15) $$\begin{array}{r} x_{id}=x_{id}+v_{id} \end{array}$$

where *f*_*i*_(*d*) denotes the dimension value of the *d*th dimension in a particle *pbest*, *f*_*i*_ = [*f*_*i*_(1), *f*_*i*_(2), …, *f*_*i*_(*D*)] denotes the learning sample vector defined for particle *i*,*pbest*_*f*_*i*_(*d*),*d*_ denotes the best position of the particle among all the particles *pbest* corresponding dimensional value. The dimension of which particle is learned depends on the parameter learning probability *Pc*. For each dimension of a particle, we generate a random number. If this random number is greater than *Pc*, the corresponding dimension will be learned from its own *pbest*. Otherwise, it will be learned in the other particle *pbest*. The algorithm selects learning particles from other particles as follows:

1. First, select two particles from the population at random, excluding the velocity update particles.
2. Then, compare the fitness values of the *pbest* of these two particles and choose the better one. In this study, the fitness value is the minimum solution of the function, which means that the smaller the function value is, the better when solving the minimization problem.

The CLPSO first assigns the learning probability *Pc* to each particle using the following equation:

(16) $$\begin{array}{r} Pc_{i}=a+b*\frac{\exp\left(\frac{10*\left(i-1\right)}{N-1}\right)}{\exp\left(10\right)-1} \end{array}$$

where a and b are two parameters used to identify the maximum and minimum learning probabilities and N is the total number of particles.

In addition to avoid wasting time in undesirable directions when the particle learns the best position of the particle individual from the sample, a particle learning count threshold m is defined and if the adaptation value of the particle does not improve after m consecutive moves, a random particle is generated again instead of the particle.

### Gray Wolf Optimizer

Mirjalili et al. (2014) proposed the metaheuristic algorithm grey wolf optimization (GWO) in 2014, a variant of the PSO with a metaphor, as proven in the recent works (Villalón et al., 2020). Similar to other metaheuristic approaches (Ala et al., 2020; Seifi et al., 2020; Moayedi and Mosavi, 2021a,b), the algorithm is inspired by the social hierarchy and hunting strategies of gray wildlife wolves and it has been applied to various problems due to its simple idea (Heidari and Pahlavani, 2017; Aljarah et al., 2019; Heidari et al., 2019; Tang et al., 2020). Regardless of its defect, we still can see some performance features in this method (Niu et al., 2019; Hu et al., 2021). In this class of methods, an initial set of agents needs to be evolved to increase the capacity to explore trends of the method within the searching process (Moayedi and Mosavi, 2021c). The best agents should be considered as alpha (α), beta (β), and delta (δ) to help other agents omega (ω) to explore more favorable areas of solution space.

In GWO, agents can identify the location of their prey and surround them. To mathematically model this behavior, the equation is as follows:

(17) $$\begin{array}{r} \vec{D}=|\vec{C}*{\vec{X}}_{p}\left(t\right)-\vec{X}\left(t\right)| \end{array}$$

(18) $$\begin{array}{r} \vec{X}\left(t+1\right)={\vec{X}}_{p}\left(t\right)-\vec{A}*\vec{D} \end{array}$$

where *t* is the number of iterations, $\vec{A}$ and $\vec{C}$ are the coefficient vectors, ${\vec{X}}_{p}$ is the position vector of the prey, and $\vec{X}$ is the position vector of the gray wolf.

$\vec{A}$ and $\vec{C\;}$ are calculated as shown below:

(19) $$\begin{array}{r} \vec{A}=2\vec{a}*{\vec{r}}_{1}-\vec{a} \end{array}$$

(20) $$\begin{array}{r} \vec{C}=2{\vec{r}}_{2} \end{array}$$

where $\vec{a}$ is decreasing from 2 to 0 with increasing number of iterations, ${\vec{r}}_{1}$ 和 ${\vec{r}}_{2}$ are random numbers between 0 and 1.

Alpha (α) agents usually lead the hunting process. So, the behavior is described by the following equation (Chantar et al., 2020):

(21) $$\begin{array}{r} {\vec{D}}_{\alpha }=|{\vec{C}}_{1}*{\vec{X}}_{\alpha }-\vec{X}| \end{array}$$

(22) $$\begin{array}{r} {\vec{D}}_{\beta }=|{\vec{C}}_{2}*{\vec{X}}_{\beta }-\vec{X}| \end{array}$$

(23) $$\begin{array}{r} {\vec{D}}_{\delta }=|{\vec{C}}_{3}*{\vec{X}}_{\delta }-\vec{X}| \end{array}$$

(24) $$\begin{array}{r} {\vec{X}}_{1}={\vec{X}}_{\alpha }-{\vec{A}}_{1}*\left({\vec{D}}_{\alpha }\right) \end{array}$$

(25) $$\begin{array}{r} {\vec{X}}_{2}={\vec{X}}_{\beta }-{\vec{A}}_{2}*\left({\vec{D}}_{\beta }\right) \end{array}$$

(26) $$\begin{array}{r} {\vec{X}}_{3}={\vec{X}}_{\delta }-{\vec{A}}_{3}*\left({\vec{D}}_{\delta }\right) \end{array}$$

(27) $$\begin{array}{r} \vec{X}\left(t+1\right)=\frac{{\vec{X}}_{1}+{\vec{X}}_{2}+{\vec{X}}_{3}}{3} \end{array}$$

### Enhanced CLPSO

The CLPSO is a well-known variant of the PSO (Fan et al., 2022), which updates the velocity of the particles by the *pbest* of all the particles, which prevents the algorithm from falling into a local optimum prematurely and prevents the algorithm from performing a local search near the global optimum. The improved algorithm GCLPSO first selects the optimal three solutions of the CLPSO algorithm, as the gray wolf algorithm alpha (α), beta (β), and delta (δ). The optimal solution of each iteration in the CLPSO algorithm is searched nearby by the GWO idea, while the searched optimal solution is substituted for the optimal solution in the CLPSO algorithm. The specific procedures of the algorithm are described as follows:

1. First, initialize the particles and parameters and calculate the fitness value of each particle
2. Update each particle using the CLPSO algorithm
3. The three optimal solutions in the CLPSO algorithm are selected as the gray wolf algorithm alpha (α), beta (β), delta (δ), and their optimal solutions are searched locally using the GWO algorithm nearby. If the optimal solution search is better than the optimal solution in the CLPSO algorithm, the optimal solution in the CLPSO algorithm will be replaced.
4. Keep looping (2), (3) steps until the termination condition is satisfied.

**Figure 3:**
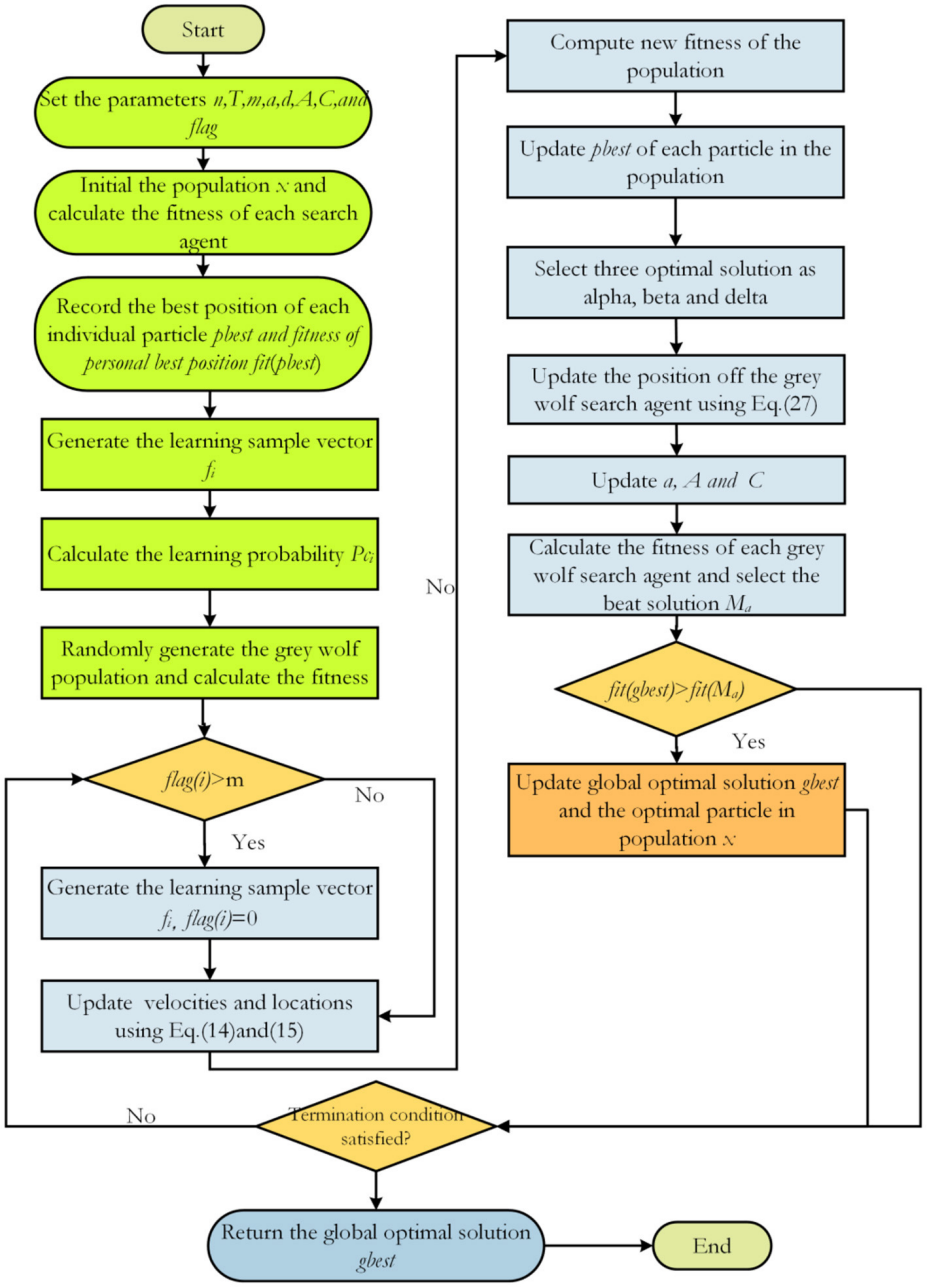
Flowchart of GCLPSO.

The flowchart of the GCLPSO algorithm is shown in Figure 3.

In the GCLPSO algorithm, *n* is the size of the population, *d* is the dimensionality, *g* is the maximum number of iterations, the population initialization is O (*n*), the gray wolf population initialization is O (*n*), the update search particle position is O (*n* × *d* × *g*), the update local search of all the gray wolf positions is O (*n* × *d* × *g*), and the sorted population fitness value is O (*n* × log *n* × *g*). Therefore, the final time complexity of the GCLPSO algorithm is 2O (*n* × *d* × *g* + *n*) + O (*n* × log *n* × *g*).

## Experiments and Results

### Image and Parameter Settings

**Figure 4:**
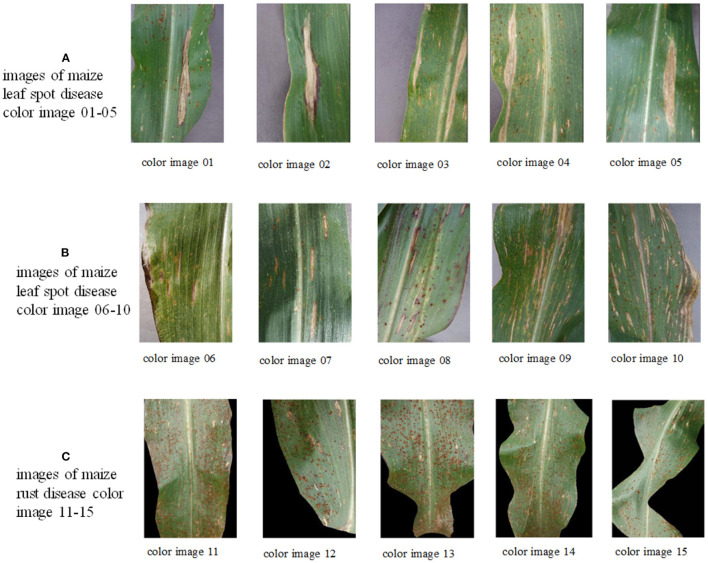
Original images of maize leaf spot disease color images 1–5, maize gray spot disease color images 6–10, and maize rust disease color images 11–15 in the Plant Village.

The experiments in this study were conducted through the maize disease image library in the Plant Village public dataset, containing images of three kinds of maize diseases: maize leaf spot, maize gray spot, and maize rust diseases and five images of each disease were taken separately, with an image size of 256 × 512 pixels. They are shown separately in Figure 4 below in (a) maize leaf spot, (b) maize gray spot, and (c) maize rust diseases.

All the experiments were conducted on a computer with a 3.40 GHz Intel® Core i7 processor and 16 GB of Random Access Memory (RAM) and programming was performed using MATLAB 2018b.

In this section of experiments, GCLPSO will be used for the practical application of multi-threshold maize disease image segmentation. To verify the effectiveness of GCLPSO on multi-threshold image segmentation, GCLPSO will be compared with CLPSO, two improved algorithms SCADE and m_SCA, and three original algorithms SSA, SCA, and SMA, respectively. To ensure the validity and fairness of the experiments (Chen et al., 2021; Moayedi and Mosavi, 2021d; Nosratabadi et al., 2021; Yang et al., 2021), all the algorithms involved in the comparisons were conducted under the same experimental conditions. Such a setting is one of the most crucial rules in the artificial intelligence community (Song et al., 2020; Thaher et al., 2020; Mousavi et al., 2021; Tavoosi et al., 2021). The population size was set to 20, the maximum number of evaluations *MaxFEs* was uniformly set to 100, and all the algorithms were tested 30 times independently to reduce the influence of random conditions. The thresholds of the experiments were set to 2, 3, and 4 thresholds, respectively. Meanwhile, the segmentation results were evaluated FSIM, PSNR, and SSIM to compare the image segmentation results, respectively. Also, we further analyzed the evaluation results of FSIM, PSNR, and SSIM using mean, variance, and the Wilcoxon signed-rank test (Liu et al., 2021a).

### Evaluation Indicators

The PSNR, SSIM index, and FSIM index is applied to further evaluate image segmentation quality (Liu et al., 2021b; Shi et al., 2021; Zhang et al., 2021).

The FSIM represents the FSIM between the original image and the segmented image. FSIM is composed of high phase composite (PC) and gradient amplitude (GM) to evaluate local structure and provide contrast information. Its value range is between 0 and 1, where the closer to 1, the better the segmentation effect. The detailed expression is as follows:

(28) $$\begin{array}{r} FSIM=\frac{\sum_{I\in \Omega }S_{L}\left(X\right)PC_{m}\left(X\right)}{\sum_{I\in \Omega }PC_{m}\left(X\right)} \end{array}$$

(29) $$\begin{array}{r} S_{L}\left(X\right)=S_{PC}\left(X\right)S_{G}\left(X\right) \end{array}$$

(30) $$\begin{array}{r} S_{PC}\left(X\right)=\frac{2PC_{1}\left(X\right)PC_{2}\left(X\right)+T_{1}}{PC_{1}^{2}\left(X\right)PC_{2}^{2}\left(X\right)+T_{1}} \end{array}$$

(31) $$\begin{array}{r} S_{G}\left(X\right)=\frac{2G_{1}\left(X\right)G_{2}\left(X\right)+T_{2}}{G_{1}^{2}\left(X\right)G_{2}^{2}\left(X\right)+T_{2}} \end{array}$$

(32) $$\begin{array}{r} G=\sqrt{G_{x}^{2}+G_{y}^{2}} \end{array}$$

(33) $$\begin{array}{r} PC\left(X\right)=\frac{E\left(X\right)}{\left(\varepsilon +\sum_{m}A_{n}\left(X\right)\right)} \end{array}$$

The Ω denotes all the pixel domains of the original image. *S*(*X*) denotes the similarity score. *P*C(*x*) means the phase consistency measure, *T*_1_ and *T*_2_ are constants, *G* is the gradient descent method, *E*(*X*) response vector size position *X* and scale *n*, furthermore, ε is a small quantity and *A*_n_(*X*) is a local scale size *n*.

The PSNR indicates the difference between the segmented image and the original image. The larger the value, the better.

It is defined as shown in Equation (34).

(34) $$\begin{array}{r} PSNR=20\cdot (\frac{255}{RMSE}) \end{array}$$

The SSIM represents a measure of the similarity of two images. The greater its value, the more effective is the segmentation of the threshold. Its definition is illustrated by Equation (35).

(35) $$\begin{array}{r} SSIM=\frac{\left(2\mu_{I}\mu_{Seg}+c_{1}\right)\left(2\sigma_{I,Seg}+c_{2}\right)}{\left({\mu_{I}}^{2}+{\mu_{Seg}}^{2}+c_{1}\right)\left({\sigma_{I}}^{2}+{\sigma_{Seg}}^{2}+c_{2}\right)} \end{array}$$

μ_I_, μ_s_, σ_I_, and σ_s_ are the mean values and SD of the original and segmented images, respectively, _S*eg*_ is the covariance of the basic image and the image segmentation, and *c*_1_ and *c*_2_ represent constants.

### Experimental Results and Analysis

In this study, in order to evaluate the image segmentation effect of GCLPSO at a multi-threshold, the comparison algorithms involved are CLPSO, SCADE, m_ SCA, SSA, SCA, and SMA. The segmentation results are evaluated using the PSNR, SSIM, and FSIM and the results are evaluated by the ways of mean, variance, and the Wilcoxon signed-rank test. Tables 2–4 show the results of the FSIM, PSNR, and SSIM comparison by the Wilcoxon signed-rank test at each threshold level of maize leaf spot disease; Tables 5–7 exhibit the results of the FSIM, PSNR, and SSIM comparison by the Wilcoxon signed-rank test at each threshold level of maize gray spot disease; Tables 8–10 show the results of the FSIM, PSNR, and SSIM comparison by the Wilcoxon signed-rank test at each threshold level of maize rust disease. Furthermore, mean means average ranking, rank means ranking order, “+” means that the performance of GCLPSO is better than the comparison algorithm, “=” means that the performance of GCLPSO is equal to the comparison algorithm, and “-” means that the performance of GCLPSO is worse than the comparison algorithm. It can be seen that GCLPSO ranks first overall and has the best performance.

**Table 2:** Results of the feature similarity FSIM comparison by the Wilcoxon signed-rank test at each threshold level of maize leaf spot disease.

**Thresholds**		**GCLPSO**	**CLPSO**	**SCADE**	**m_SCA**	**SSA**	**SCA**	**SMA**
2	+/-/=	~	1/0/4	1/0/4	3/0/2	5/0/0	5/0/0	5/0/0
	Mean	**1.2**	2	3	5.2	8.2	9.8	7.8
	Rank	**1**	2	3	5	6	7	5
3	+/-/=	~	1/0/4	2/1/2	5/0/0	5/0/0	5/0/0	5/0/0
	Mean	**1.6**	2.6	3.4	5.8	8.8	9.6	8.4
	Rank	**1**	2	3	4	6	7	5
4	+/-/=	~	0/0/5	1/0/4	3/0/2	4/0/1	5/0/0	2/0/3
	Mean	**2.2**	5.2	4.6	5.8	9.2	9.8	7
	Rank	**1**	3	2	4	6	7	5

*Mean, means the mean value of the ranking of the SSIM evaluation results obtained by the algorithm after segmentation of each image at the 2 threshold level, Rank, means the overall ranking*.

**Table 3:** Results of the peak signal-to-noise ratio (PSNR) comparison by the Wilcoxon signed-rank test at each threshold level of maize leaf spot disease.

**Thresholds**		**GCLPSO**	**CLPSO**	**SCADE**	**m_SCA**	**SSA**	**SCA**	**SMA**
2	+/-/=	~	0/0/5	1/0/4	3/0/2	4/0/1	5/0/0	3/0/2
	Mean	**1.4**	2	2.6	5.6	8.6	8.4	7.2
	Rank	**1**	2	3	4	7	6	5
3	+/-/=	~	1/0/4	1/0/4	4/0/1	5/0/0	5/0/0	5/0/0
	Mean	**1.8**	2.8	3.4	5.2	8.8	8.6	8.4
	Rank	**1**	2	3	4	7	6	5
4	+/-/=	~	1/0/4	1/0/4	3/0/2	4/0/1	5/0/0	3/0/2
	Mean	**2.4**	4.4	5.6	6	9	9.4	7
	Rank	**1**	2	3	4	6	7	5

*Mean, means the mean value of the ranking of the SSIM evaluation results obtained by the algorithm after segmentation of each image at the 2 threshold level, Rank, means the overall ranking*.

**Table 4:** Results of the structural similarity (SSIM) comparison by the Wilcoxon signed-rank test at each threshold level of maize leaf spot disease.

**Thresholds**		**GCLPSO**	**CLPSO**	**SCADE**	**m_SCA**	**SSA**	**SCA**	**SMA**
2	+/-/=	~	0/0/5	2/0/3	3/0/2	4/0/1	5/0/0	3/0/2
	Mean	**1.2**	2	3	5.2	8.8	7.8	7
	Rank	**1**	2	3	4	8	7	5
3	+/-/=	~	0/0/5	1/0/4	4/0/1	5/0/0	5/0/0	5/0/0
	Mean	**1.8**	3.2	3.2	4.8	8.2	8.6	7.8
	Rank	**1**	2	2	3	5	6	4
4	+/-/=	~	1/0/4	1/0/4	2/0/3	4/0/1	5/0/0	2/0/3
	Mean	**2.6**	5.6	4.4	5.2	9	9.4	6.8
	Rank	**1**	4	2	3	6	7	5

*Mean, means the mean value of the ranking of the SSIM evaluation results obtained by the algorithm after segmentation of each image at the 2 threshold level, Rank, means the overall ranking*.

**Table 5:** Results of the FSIM comparison by the Wilcoxon signed-rank test at each threshold level of maize gray spot disease.

**Thresholds**		**GCLPSO**	**CLPSO**	**SCADE**	**m_SCA**	**SSA**	**SCA**	**SMA**
2	+/-/=	~	0/0/5	1/0/4	4/0/1	5/0/0	5/0/0	5/0/0
	Mean	**1.4**	2	3.2	5.6	6.4	10	9
	Rank	**1**	2	3	4	5	7	6
3	+/-/=	~	1/0/4	1/0/4	2/0/3	5/0/0	5/0/0	5/0/0
	Mean	**2.2**	3.4	2.2	6	9.4	9.2	7.6
	Rank	**1**	2	1	3	6	5	4
4	+/-/=	~	1/0/4	1/0/4	2/0/3	5/0/0	5/0/0	3/0/2
	Mean	**3**	5.4	4	6.8	9.2	9.8	6.8
	Rank	**1**	3	2	4	5	6	4

*Mean, means the mean value of the ranking of the SSIM evaluation results obtained by the algorithm after segmentation of each image at the 2 threshold level, Rank, means the overall ranking*.

**Table 6:** Results of the PSNR comparison by the Wilcoxon signed-rank test at each threshold level of maize gray spot disease.

**Thresholds**		**GCLPSO**	**CLPSO**	**SCADE**	**m_SCA**	**SSA**	**SCA**	**SMA**
2	+/-/=	~	0/0/5	1/0/4	3/0/2	3/0/2	5/0/0	5/0/0
	Mean	**1.4**	2.4	3.4	5.2	6.8	9.4	9.2
	Rank	**1**	2	3	4	5	7	6
3	+/-/=	~	1/0/4	2/0/3	2/0/3	4/0/1	3/0/2	3/0/2
	Mean	**2.6**	3	3.6	5.6	9.6	8.6	7.2
	Rank	**1**	2	3	4	8	7	5
4	+/-/=	~	1/0/4	1/0/4	1/0/4	3/0/2	2/0/3	2/0/3
	Mean	**3.4**	3.8	4.8	5.8	9.4	9	6.6
	Rank	**1**	2	3	4	7	6	5

*Mean, means the mean value of the ranking of the SSIM evaluation results obtained by the algorithm after segmentation of each image at the 2 threshold level, Rank, means the overall ranking*.

**Table 7:** Results of the SSIM comparison by the Wilcoxon signed-rank test at each threshold level of maize gray spot disease.

**Thresholds**		**GCLPSO**	**CLPSO**	**SCADE**	**m_SCA**	**SSA**	**SCA**	**SMA**
2	+/-/=	~	0/0/5	1/0/4	4/0/1	4/0/1	5/0/0	5/0/0
	Mean	**1.8**	2.6	2.8	5.2	7.2	9.4	9.2
	Rank	**1**	2	3	4	5	7	6
3	+/-/=	~	1/0/4	2/0/3	2/0/3	4/0/1	5/0/0	4/0/1
	Mean	**2.4**	3.2	3	5.4	9.6	8.8	7.4
	Rank	**1**	3	2	4	7	6	5
4	+/-/=	~	0/0/5	1/0/4	1/0/4	4/0/1	3/0/2	2/0/3
	Mean	**3.6**	4	4.4	6.4	9.4	9.4	6.4
	Rank	**1**	2	3	4	5	5	4

*Mean, means the mean value of the ranking of the SSIM evaluation results obtained by the algorithm after segmentation of each image at the 2 threshold level, Rank, means the overall ranking*.

**Table 8:** Results of the FSIM comparison by the Wilcoxon signed-rank test at each threshold level of maize rust disease.

**Thresholds**		**GCLPSO**	**CLPSO**	**SCADE**	**m_SCA**	**SSA**	**SCA**	**SMA**
2	+/-/=	~	0/1/4	1/0/4	1/0/4	1/0/4	4/0/1	4/0/1
	Mean	**3.6**	3.8	4.2	5.6	5.8	9.2	9
	Rank	**1**	2	3	4	5	7	6
3	+/-/=	~	2/0/3	4/0/1	3/0/2	5/0/0	5/0/0	5/0/0
	Mean	**1**	3.2	6.4	4.4	8.6	9.2	8.6
	Rank	**1**	2	4	3	5	6	5
4	+/-/=	~	1/0/4	2/0/3	2/0/3	4/0/1	5/0/0	4/0/1
	Mean	**2**	5.6	6.8	3.8	8.4	9.4	7.8
	Rank	**1**	3	4	2	6	7	5

*Mean, means the mean value of the ranking of the SSIM evaluation results obtained by the algorithm after segmentation of each image at the 2 threshold level, Rank, means the overall ranking*.

**Table 9:** Results of the PSNR comparison by the Wilcoxon signed-rank test at each threshold level of maize rust disease.

**Thresholds**		**GCLPSO**	**CLPSO**	**SCADE**	**m_SCA**	**SSA**	**SCA**	**SMA**
2	+/-/=	**~**	0/1/4	1/1/3	1/0/4	1/0/4	4/0/1	4/0/1
	Mean	**4.4**	4.4	3.6	5.6	5.6	9.6	8.8
	Rank	**1**	1	1	3	3	5	4
3	+/-/=	~	1/0/4	4/0/1	3/0/2	5/0/0	5/0/0	5/0/0
	Mean	**1.2**	3.2	6.6	4.8	8.6	9.2	8.6
	Rank	**1**	2	4	3	5	6	5
4	+/-/=	~	1/0/4	2/0/3	2/0/3	4/0/1	5/0/0	5/0/0
	Mean	**1.6**	6	6.2	4.2	7.8	9.4	8
	Rank	**1**	3	4	2	5	7	6

*Mean, means the mean value of the ranking of the SSIM evaluation results obtained by the algorithm after segmentation of each image at the 2 threshold level, Rank, means the overall ranking*.

**Table 10:** Results of the SSIM comparison by the Wilcoxon signed-rank test at each threshold level of maize rust disease.

**Thresholds**		**GCLPSO**	**CLPSO**	**SCADE**	**m_SCA**	**SSA**	**SCA**	**SMA**
2	+/-/=	~	0/1/4	1/0/4	1/0/4	1/0/4	5/0/0	4/0/1
	Mean	**4.4**	4.4	4.8	5.2	5.6	9.6	8.8
	Rank	**1**	1	2	3	4	6	5
3	+/-/=	~	1/0/4	4/0/1	3/0/2	5/0/0	5/0/0	5/0/0
	Mean	**1.2**	3	6.4	5	8.4	9.2	8.6
	Rank	**1**	2	4	3	5	7	6
4	+/-/=	~	2/0/3	2/0/3	2/0/3	4/0/1	5/0/0	4/0/1
	Mean	**1.6**	6.2	6.2	4	8	9.4	8
	Rank	**1**	3	3	2	4	5	4

*Mean, means the mean value of the ranking of the SSIM evaluation results obtained by the algorithm after segmentation of each image at the 2 threshold level, Rank, means the overall ranking*.

**Figure 5:**
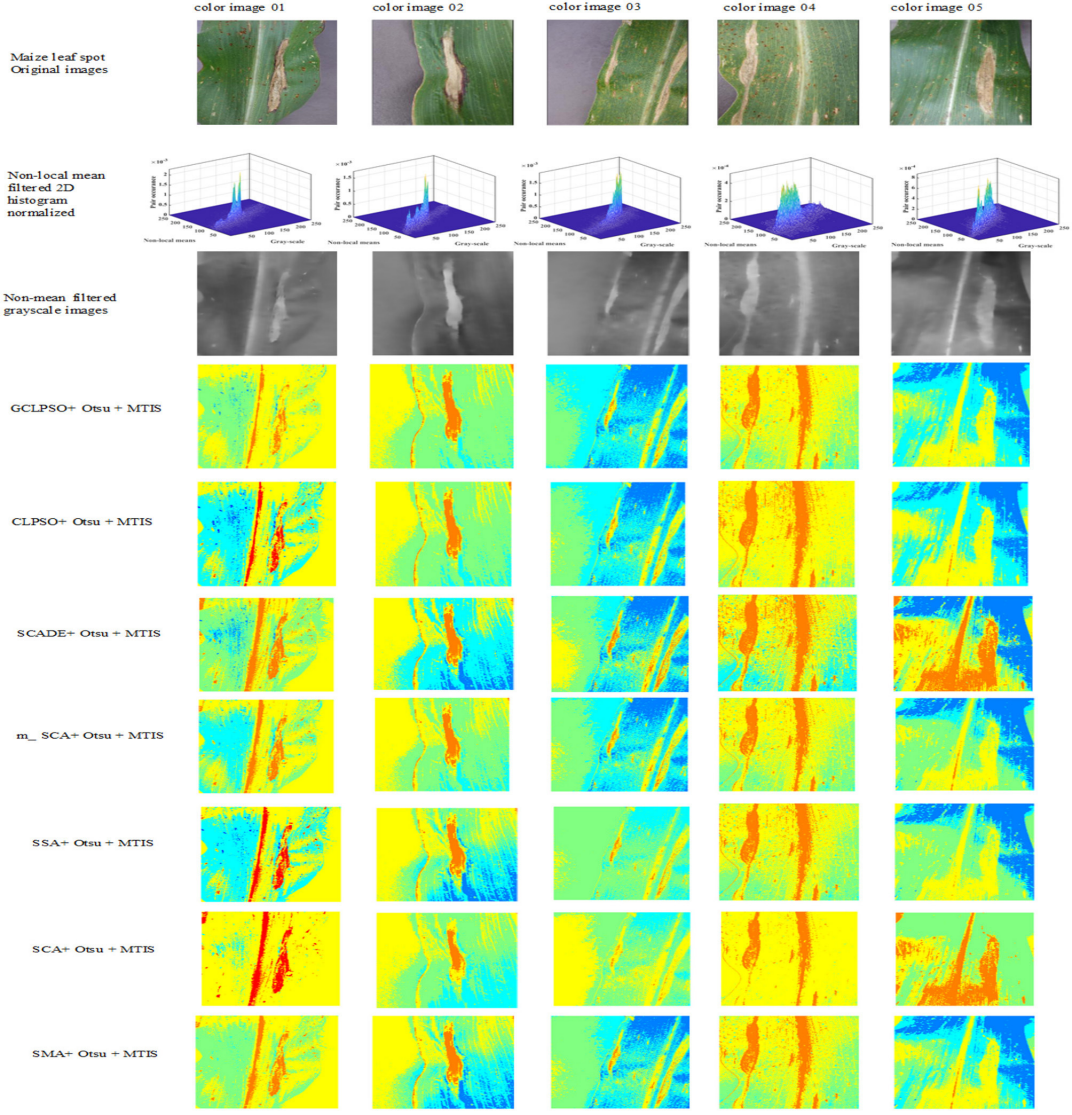
Original images, non-local mean filtered normalized 2D histogram, non-local mean filtered grayscale image results, and four-threshold color image segmentation results of all the algorithms of maize leaf spot disease color images 1–5.

**Figure 6:**
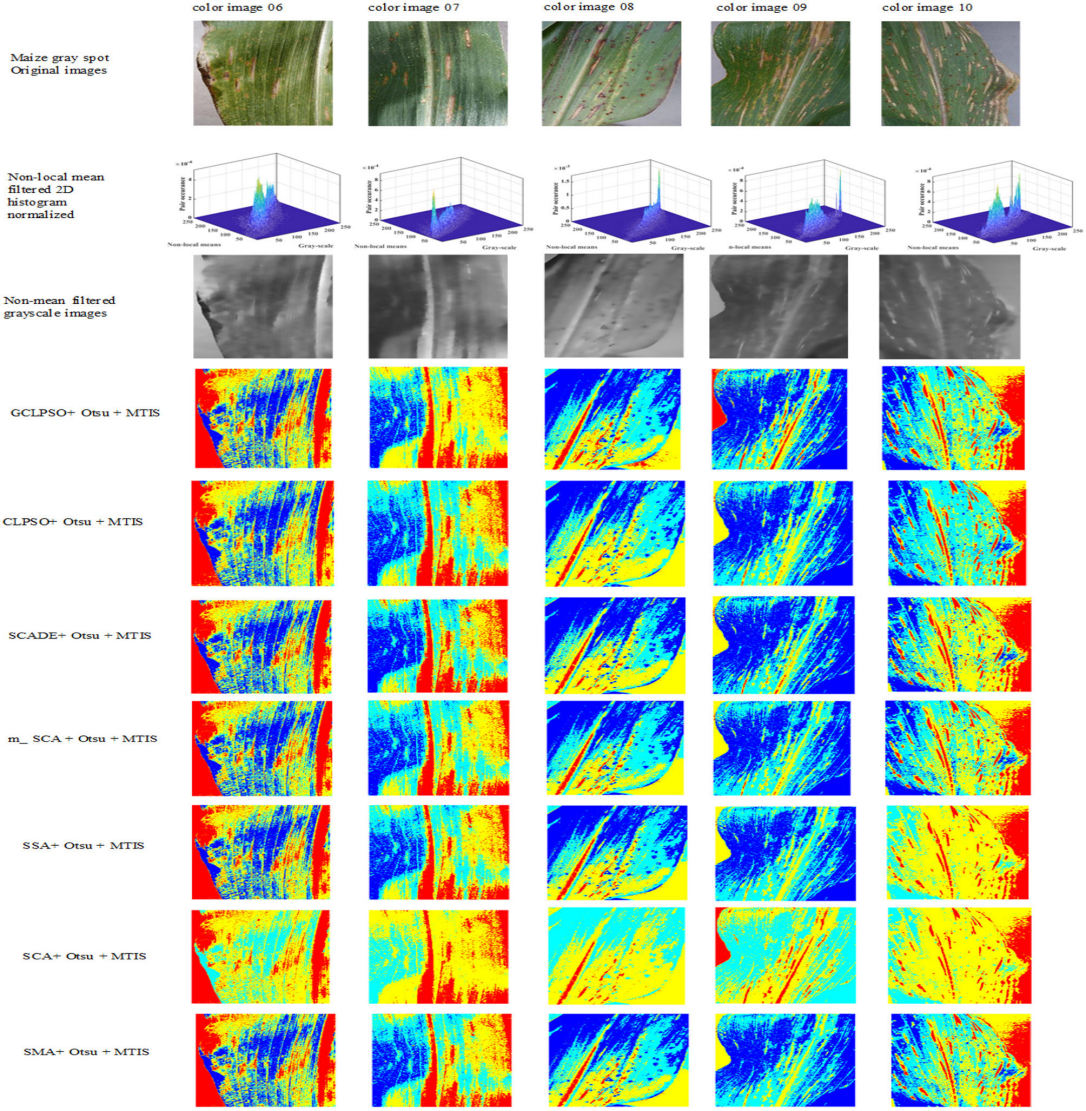
Original images, non-local mean filtered normalized 2D histogram, non-local mean filtered grayscale image results, and three-threshold color image segmentation results of all the algorithms of maize gray spot disease color images 6–10.

**Figure 7:**
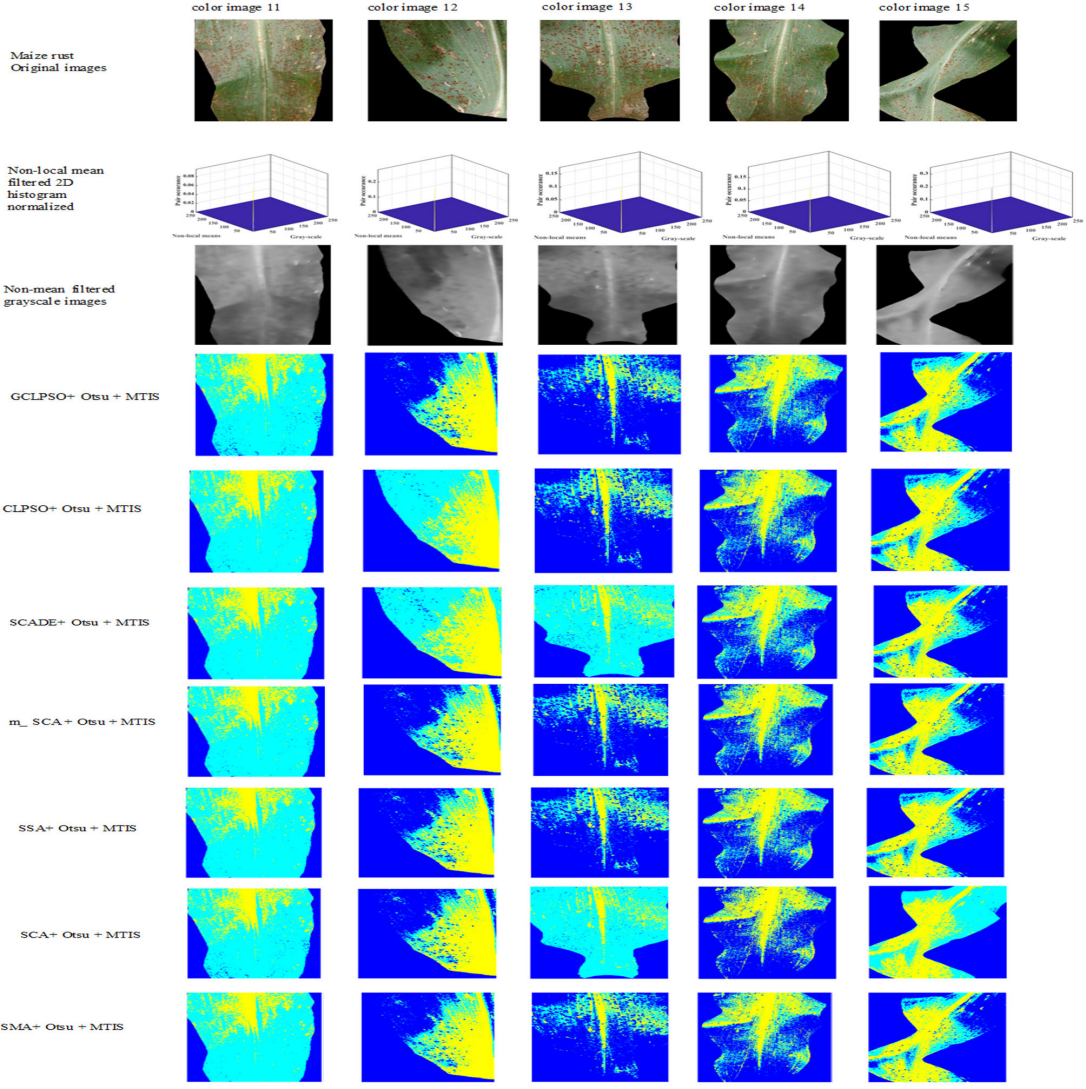
Original images, non-local mean filtered 2D histogram, non-local mean filtered grayscale image results, and two-threshold color image segmentation results of all the algorithms of maize rust disease color images 11–15.

In the experience on MTIS, images 1–5 are maize leaf spot disease, images 6–10 are maize gray spot disease, and images 11–15 are maize rust disease. The image size was set to 256 × 512 pixels. Figure 5 shows that the first row presents the original images of five randomly selected color images of corn leaf spot disease in the Plant village public dataset; the second row presents the grayscale and non-mean filtered normalized 3D histogram of the corresponding image, with the X-axis as the gray value of the grayscale image, the Y-axis as the gray value of the non-mean filtered image and the Z-axis as the normalized result of the combination of the gray value of the grayscale image and the non-mean filtered image; the third row is the non-local mean filtered grayscale image of the corresponding image; the remaining rows are the color segmentation results of the GCLPSO algorithm combined with the non-local mean filter and the contrast algorithm to optimize the Otsu segmentation with four-threshold, respectively. Figure 6 denotes the original images; non-local mean filtered normalized 2D histogram, non-local mean filtered grayscale image results, and three-threshold color image segmentation results of all the maize gray spot disease algorithms—color images 6–10. Figure 7 is similar to Figures 5, 6 for the two-threshold color segmentation results of all the corn rust spot disease algorithms—color images 11–15. We can see that the segmentation effect under the GCLPSO algorithm is significantly better than other similar algorithms.

**Figure 8:**
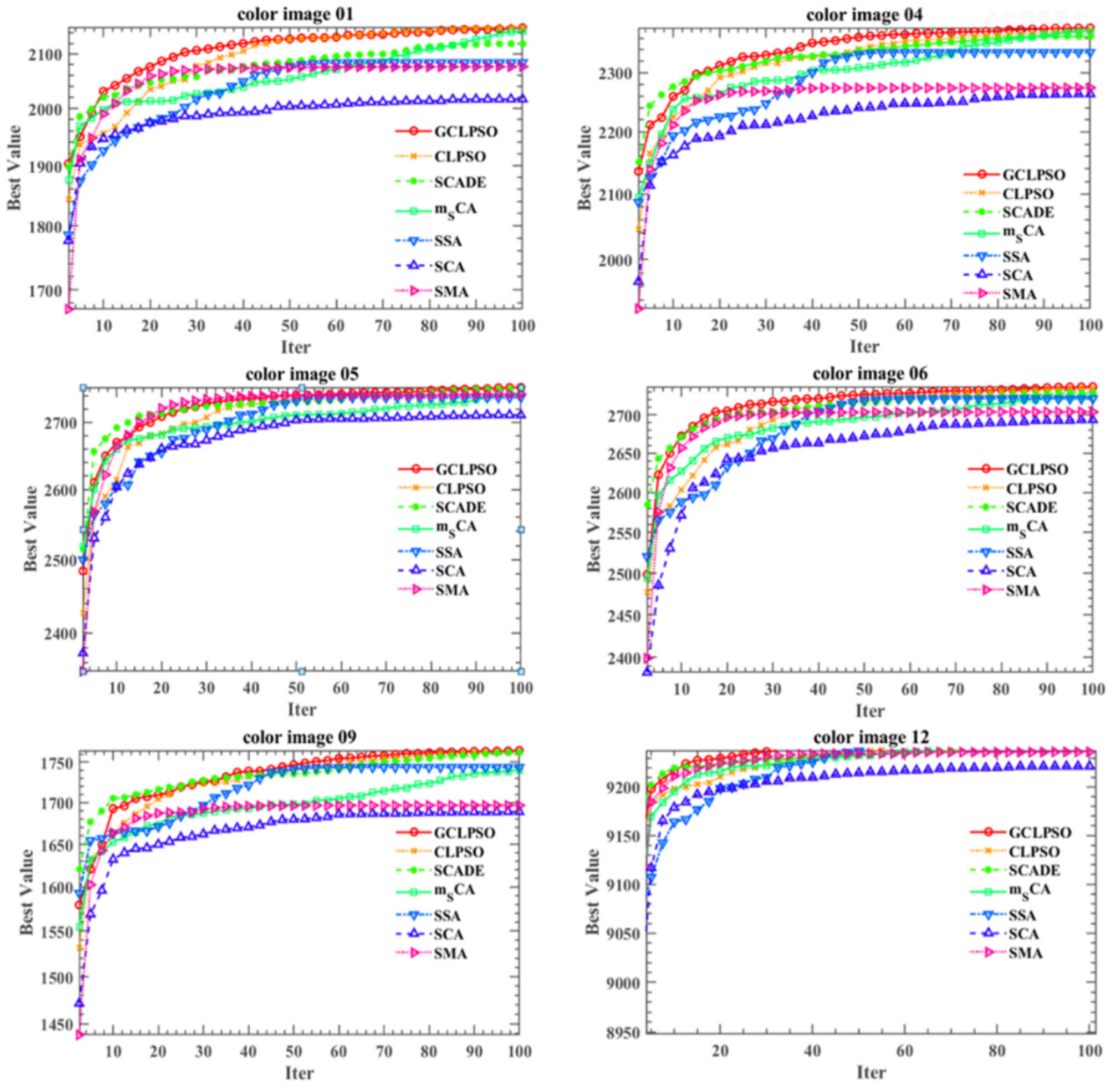
Convergence curves of 2D-Otsu at threshold 2.

According to Figure 8, the local features of the image acquired by GCLPSO are obviously better than those of other algorithms. It is easy to conclude that GCLPSO surpasses other competing algorithms at the threshold level of 2.

### Conclusion

**Figure 9:**
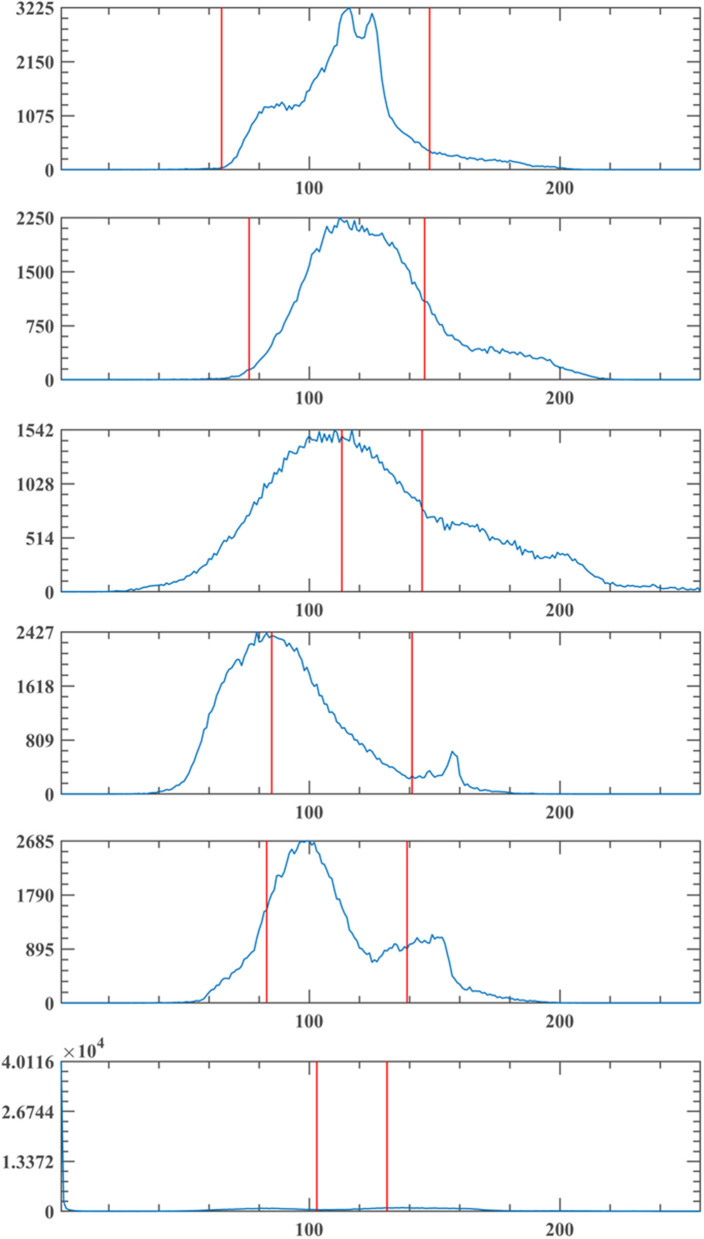
Segmentation threshold line of GCLPSO-2D-Otsu at threshold 2.

Figures 5–7 show the segmentation results under different algorithms; from the segmentation results, the local features of the images retained by GCLPSO are better than those retained by other algorithms. From the data, Tables 2–10 show the comparison of mean and SD of FSIM index, PSNR, and SSIM index for all the algorithms segmentation of maize leaf spot, gray spot, and rust images. Appendix Tables 11–19 show the results of FSIM index, PSNR, and SSIM index comparison by the Wilcoxon signed-rank test at each threshold level for maize leaf spot, gray spot, and rust images. The comparison results showed that the feature similarity index outperformed other algorithms at thresholds 2, 3, and 4 for all the disease images and also performed significantly better than other algorithms at other thresholds. Figure 8 shows the convergence curves of images 1, 4, 5, 6, 9, and 12 when the image segmentation experiments were performed at a threshold level of 2, 3, and 4. Based on the convergence curves, it can be seen that GCLPSO is better at finding the maximum value of the maximum interclass variance and it has higher convergence accuracy than other algorithms. Therefore, based on the above comparison and experimental analysis, GCLPSO outperformed the other algorithms at the level of multiple thresholds for maize with multiple disease spots. The threshold values and segmentation threshold lines selected by GCLPSO for the 2-threshold segmentation of maize disease image (01)(04)(06)(09)(10)(12) can be seen in Figure 9 above, and it can be seen that the threshold values were selected more reasonably.

## Summary and Future Work

According to the multi-threshold image segmentation experiments on three maize disease images, the used GCLPSO was compared with CLPSO and two other improved algorithms and three original algorithms, respectively, and the multi-threshold segmentation comparison experiments were compared with each other at the same time. In addition, the PSNR, SSIM, and FSIM were used to compare the image segmentation results for evaluation and the evaluation results were analyzed using the mean, variance, and the Wilcoxon signed-rank tests. It can be seen from the experiments in this study that the GCLPSO algorithm can be used as an optimization tool for the Otsu segmentation of maize disease images in multi-threshold level segmentation results by non-local average filtered 2D histogram using an improved swarm intelligent optimization algorithm since GCLPSO can obtain the best selection of thresholds and has ideal stability in the segmentation process. Therefore, it can be effectively used in maize leaf disease image segmentation.

In future research work, the GCLPSO can be combined with other optimization methods for image segmentation of multicrop diseases to improve the identification and intelligent diagnosis of disease deficiency in maize and other crops and effectively reduce the economic losses caused by crop diseases.

(a) Maize leaf spot disease

Maize leaf spot disease, also known as stripe disease, coal stripe disease, leaf blight disease, and large spot disease, is a major foliar disease of maize, which occurs throughout China and causes heavy damage. It mainly affects the leaves, the leaf sheaths, and bracts in severe cases. Generally, from the bottom of the leaf first, it gradually expands upward, when serious spots can spread throughout the plant, but there are also from the upper leaves of the case. Infected leaves form large nucleate spots, which are initially water-stained greenish-gray or grayish-green spots in the field and then expand into large diamond-shaped or long fusiform spots with dark brown margins and light brown or gray centers, generally 5–10 cm long, with a distinct black-brown mold layer on the spots when wet, and in severe cases, the spots combine to split longitudinally, and the leaves die.

(b) Maize gray spot disease

Maize gray spot disease, also known as tail spore leaf spot disease and corn mold spot disease, is one of the diseases that have been rising rapidly and causing more serious damage in the recent years. It mainly affects the leaves. At first, on the leaf surface to form no obvious edge of the oval, moment round gray to light brown spots, later turned brown. The spots are mostly limited to between the parallel leaf veins, size 4–20 × 2–5 (mm). When the humidity is high, the back of the spot produces gray moldy material, i.e., the conidiophore and conidia of the disease. Sometimes, it causes the fruiting spike to rot or droop and the seeds to blacken.

(c) Maize rust spot disease

Maize rust mainly affects the leaves, but it can also occur on the cob bracts and male flowers in severe cases. The upper and middle leaves of the plant are heavily affected, initially with inconspicuous yellowish dots scattered or clustered on the adaxial surface of the leaves, later protruding and expanding into a round to oblong, yellowish brown or brown, with the surrounding epidermis turning up and scattering rust-colored powder (summer spores of the pathogenic fungus). Later on, the spot grows round black protrusions, rupture to reveal black-brown powder (winter spores of the pathogenic fungus). The disease is caused by the fungus, the field leaf disease, the disease produced by the summer spores spread by airflow, reinfestation, spread, and expansion. Production of early maturing varieties is susceptible to the disease, heavy incidence of biased nitrogen fertilization, high temperature, humidity, rain, foggy days, and insufficient light facilitate the prevalence of maize rust (Lv et al., 2020).

## Data Availability Statement

The datasets presented in this study can be found in online repositories. This data can be found here: https://github.com/Tmcsn/AI-Challenger-2018-CropDisease.

## Author Contributions

CC and AH: writing—original draft, writing—review and editing, software, visualization, and investigation. XW, HC, and HY: conceptualization, methodology, formal analysis, investigation, writing—review and editing, funding acquisition, and supervision. All authors contributed to the article and approved the submitted version.

## Funding

This research was supported by Science and Technology Development Project of Jilin Province (20190301024NY).

## Conflict of Interest

The authors declare that the research was conducted in the absence of any commercial or financial relationships.

## Publisher's Note

All claims expressed in this article are solely those of the authors and do not necessarily represent those of their affiliated organizations, or those of the publisher, the editors and the reviewers. Any product that may be evaluated in this article, or claim that may be made by its manufacturer, is not guaranteed or endorsed by the publisher.

## Footnotes

^1^Available online at: https://github.com/Tmcsn/AI-Challenger-2018-CropDisease.
