# Supplementary material for: Multi-Threshold Image Segmentation of Maize Diseases Based on Elite Comprehensive Particle Swarm Optimization and Otsu
Source: Front Plant Sci. 2021 Dec 13;12:789911. doi: 10.3389/fpls.2021.789911 (PMC8710579; doi:10.3389/fpls.2021.789911)
Supplement: Supplementary file 1 [file Data_Sheet_1.docx]

# Appendix

In this part, Tables 1 to 3 show the comparison of mean and SD of the FSIM, PSNR, and SSIM of all the algorithms for segmentation of maize leaf spot images—color images 1–5; Tables 4 to 6 show the comparison of mean and SD of the FSIM, PSNR, and SSIM of all the algorithms for segmentation of maize gray spot images—color images 6–10; and Tables 7 to 9 show the comparison of mean and SD of the FSIM, PSNR, and SSIM of all the algorithms for segmentation of maize rust disease images—color images 11–15.

**Table 1**. Comparison of mean and SD of the FSIM of all the algorithms for segmentation of maize leaf spot images color image 1–5.

| Image | Thresholds | Item | GCLPSO | CLPSO | SCADE | m_ SCA | SSA | SCA | SMA |
| --- | --- | --- | --- | --- | --- | --- | --- | --- | --- |
| color image 01 | 2 | AVG | **6.1476E-01** | 6.0918E-01 | 6.0602E-01 | 6.0864E-01 | 5.8822E-01 | 5.8780E-01 | 5.9569E-01 |
|  |  | STD | 1.1375E-02 | **1.0211E-02** | 1.4413E-02 | 1.1617E-02 | 2.2726E-02 | 1.7952E-02 | 2.3296E-02 |
|  | 3 | AVG | **6.7760E-01** | 6.5495E-01 | 6.5453E-01 | 6.4288E-01 | 6.0484E-01 | 6.0147E-01 | 6.1146E-01 |
|  |  | STD | 3.5721E-02 | 4.2200E-02 | 4.0782E-02 | 5.1947E-02 | 3.6302E-02 | **1.9286E-02** | 3.0628E-02 |
|  | 4 | AVG | 6.7074E-01 | 6.6780E-01 | 6.6489E-01 | 6.7053E-01 | 6.2886E-01 | 6.0768E-01 | 6.4741E-01 |
|  |  | STD | 4.7175E-02 | 4.7081E-02 | 4.7614E-02 | 6.4190E-02 | 4.5034E-02 | **3.6452E-02** | 5.2247E-02 |
| color image 02 | 2 | AVG | **5.8198E-01** | 5.8034E-01 | 5.8046E-01 | 5.7838E-01 | 5.7368E-01 | 5.6287E-01 | 5.7400E-01 |
|  |  | STD | 7.7172E-03 | 1.0341E-02 | 1.2097E-02 | **6.8942E-03** | 1.5259E-02 | 1.7755E-02 | 1.5540E-02 |
|  | 3 | AVG | **6.4262E-01** | 6.2565E-01 | 5.9474E-01 | 6.1290E-01 | 6.0805E-01 | 5.7421E-01 | 5.8363E-01 |
|  |  | STD | 2.3455E-02 | 3.6221E-02 | 3.7731E-02 | 3.9647E-02 | 4.6359E-02 | 2.3679E-02 | **1.6966E-02** |
|  | 4 | AVG | 6.4933E-01 | 6.3724E-01 | 6.1613E-01 | 6.2232E-01 | 5.9403E-01 | 5.9086E-01 | 6.2901E-01 |
|  |  | STD | **3.6479E-02** | 3.8359E-02 | 4.8355E-02 | 4.6389E-02 | 4.5094E-02 | 3.6754E-02 | 4.8979E-02 |
| color image 03 | 2 | AVG | **6.0138E-01** | 5.9229E-01 | 5.9057E-01 | 5.5728E-01 | 5.4401E-01 | 5.2241E-01 | 5.4443E-01 |
|  |  | STD | **2.5109E-02** | 3.4190E-02 | 3.9767E-02 | 5.0290E-02 | 4.6144E-02 | 4.0741E-02 | 5.1001E-02 |
|  | 3 | AVG | **6.3147E-01** | 6.2103E-01 | 6.2145E-01 | 5.9520E-01 | 5.7500E-01 | 5.5454E-01 | 5.8014E-01 |
|  |  | STD | 3.9494E-02 | **3.7927E-02** | 6.6049E-02 | 5.6942E-02 | 5.6374E-02 | 4.8696E-02 | 5.7830E-02 |
|  | 4 | AVG | **6.6037E-01** | 6.4083E-01 | 6.5746E-01 | 6.1918E-01 | 6.0125E-01 | 5.8342E-01 | 6.2880E-01 |
|  |  | STD | 5.1013E-02 | 5.0494E-02 | 4.3896E-02 | 6.1069E-02 | 5.9326E-02 | 5.4995E-02 | 5.6571E-02 |
| color image 04 | 2 | AVG | 5.7339E-01 | **5.7580E-01** | 5.6550E-01 | 5.4209E-01 | 5.1667E-01 | 4.8648E-01 | 4.8863E-01 |
|  |  | STD | 1.9956E-02 | **1.5091E-02** | 5.2009E-02 | 5.2695E-02 | 6.0586E-02 | 6.0667E-02 | 5.7671E-02 |
|  | 3 | AVG | 6.4953E-01 | 6.2752E-01 | 6.3768E-01 | 5.9264E-01 | 5.3139E-01 | 5.7566E-01 | 5.6388E-01 |
|  |  | STD | 6.1191E-02 | 6.5504E-02 | 7.0803E-02 | 7.2491E-02 | 7.8705E-02 | **5.3094E-02** | 7.0870E-02 |
|  | 4 | AVG | 6.5898E-01 | 6.2661E-01 | 6.5043E-01 | 6.5481E-01 | 6.2114E-01 | 5.7832E-01 | 6.3107E-01 |
|  |  | STD | 6.7859E-02 | 5.7298E-02 | 6.4633E-02 | 7.4147E-02 | 7.4134E-02 | **4.3925E-02** | 7.8303E-02 |
| color image 05 | 2 | AVG | **5.8178E-01** | 5.7715E-01 | 5.7391E-01 | 5.4151E-01 | 5.2653E-01 | 5.2440E-01 | 5.3878E-01 |
|  |  | STD | **9.8114E-03** | 2.0893E-02 | 3.1026E-02 | 3.9391E-02 | 2.8097E-02 | 3.5516E-02 | 3.3156E-02 |
|  | 3 | AVG | 6.0330E-01 | 6.1477E-01 | **6.2175E-01** | 5.7575E-01 | 5.4702E-01 | 5.3387E-01 | 5.5855E-01 |
|  |  | STD | 3.3591E-02 | **2.4817E-02** | 3.9976E-02 | 4.1424E-02 | 4.2071E-02 | 4.0982E-02 | 2.8922E-02 |
|  | 4 | AVG | 6.3120E-01 | 6.2639E-01 | **6.3607E-01** | 5.9925E-01 | 5.6852E-01 | 5.7484E-01 | 5.7675E-01 |
|  |  | STD | 3.2432E-02 | 4.0713E-02 | **2.5229E-02** | 4.1235E-02 | 4.0706E-02 | 3.4220E-02 | 3.7125E-02 |

**Table 2**. Comparison of mean and SD of the PSNR for all the algorithms segmentation of maize leaf spot images color images 1–5.

| Image | Thresholds | Item | GCLPSO | CLPSO | SCADE | m_ SCA | SSA | SCA | SMA |
| --- | --- | --- | --- | --- | --- | --- | --- | --- | --- |
| color image 01 | 2 | AVG | **1.5166E+01** | 1.5250E+01 | 1.5083E+01 | 1.5005E+01 | 1.3147E+01 | 1.3308E+01 | 1.3759E+01 |
|  |  | STD | 6.0626E-01 | **9.6919E-01** | 1.0121E+00 | 7.6413E-01 | 2.5373E+00 | 2.2497E+00 | 2.6616E+00 |
|  | 3 | AVG | **1.9130E+01** | 1.8088E+01 | 1.8241E+01 | 1.6946E+01 | 1.4207E+01 | 1.4556E+01 | 1.5199E+01 |
|  |  | STD | 2.1002E+00 | 2.2584E+00 | 2.6850E+00 | 3.3787E+00 | 3.1877E+00 | **1.8051E+00** | 1.8694E+00 |
|  | 4 | AVG | 1.8822E+01 | 1.8619E+01 | 1.8381E+01 | 1.8339E+01 | 1.6213E+01 | 1.4982E+01 | 1.7316E+01 |
|  |  | STD | 2.5004E+00 | 2.6862E+00 | 3.1211E+00 | 3.5959E+00 | 2.8052E+00 | **2.5944E+00** | 3.0521E+00 |
| color image 02 | 2 | AVG | **1.4855E+01** | 1.4492E+01 | 1.4553E+01 | 1.4007E+01 | 1.3867E+01 | 1.3827E+01 | 1.4471E+01 |
|  |  | STD | 1.3020E+00 | 1.5770E+00 | 1.4908E+00 | **1.9084E+00** | 2.0318E+00 | 1.4291E+00 | 1.6522E+00 |
|  | 3 | AVG | **1.8137E+01** | 1.7269E+01 | 1.4964E+01 | 1.6687E+01 | 1.6381E+01 | 1.3991E+01 | 1.4691E+01 |
|  |  | STD | 1.3483E+00 | 2.4786E+00 | 1.7401E+00 | 2.1089E+00 | 2.5922E+00 | 2.0947E+00 | **1.8947E+00** |
|  | 4 | AVG | 1.8563E+01 | 1.7773E+01 | 1.6345E+01 | 1.6582E+01 | 1.4949E+01 | 1.4814E+01 | 1.6918E+01 |
|  |  | STD | **1.7937E+00** | 2.5962E+00 | 2.9051E+00 | 3.0867E+00 | 2.8556E+00 | 2.5914E+00 | 3.2253E+00 |
| color image 03 | 2 | AVG | **1.5420E+01** | 1.5282E+01 | 1.5177E+01 | 1.4735E+01 | 1.3599E+01 | 1.4805E+01 | 1.3814E+01 |
|  |  | STD | **3.3438E-01** | 4.9631E-01 | 4.0495E-01 | 1.5215E+00 | 3.0646E+00 | 8.0416E-01 | 2.7700E+00 |
|  | 3 | AVG | **1.6291E+01** | 1.5820E+01 | 1.5835E+01 | 1.5358E+01 | 1.4786E+01 | 1.5169E+01 | 1.4931E+01 |
|  |  | STD | 1.3657E+00 | **1.3925E+00** | 1.4004E+00 | 6.6211E-01 | 2.1507E+00 | 1.0983E+00 | 1.6155E+00 |
|  | 4 | AVG | **1.6739E+01** | 1.6388E+01 | 1.6380E+01 | 1.5581E+01 | 1.4752E+01 | 1.5076E+01 | 1.5858E+01 |
|  |  | STD | 1.9178E+00 | 2.4434E+00 | 1.4157E+00 | 1.8455E+00 | 2.6414E+00 | 2.5177E+00 | 9.6084E-01 |
| color image 04 | 2 | AVG | 1.5958E+01 | **1.6081E+01** | 1.5221E+01 | 1.4111E+01 | 1.3097E+01 | 1.0601E+01 | 1.1713E+01 |
|  |  | STD | 6.2872E-01 | **4.4336E-01** | 2.9615E+00 | 3.4586E+00 | 3.6084E+00 | 3.8273E+00 | 3.5904E+00 |
|  | 3 | AVG | 1.8523E+01 | 1.7538E+01 | 1.7847E+01 | 1.6357E+01 | 1.3999E+01 | 1.5742E+01 | 1.5188E+01 |
|  |  | STD | 1.9841E+00 | 2.2753E+00 | 2.6754E+00 | 2.9269E+00 | 3.6081E+00 | **2.5429E+00** | 3.3103E+00 |
|  | 4 | AVG | 1.8641E+01 | 1.7318E+01 | 1.8491E+01 | 1.8748E+01 | 1.7496E+01 | 1.5915E+01 | 1.7810E+01 |
|  |  | STD | 2.6927E+00 | 2.2370E+00 | 2.3012E+00 | 2.3899E+00 | 2.2468E+00 | **2.1407E+00** | 2.4063E+00 |
| color image 05 | 2 | AVG | **1.4856E+01** | 1.4359E+01 | 1.4466E+01 | 1.2402E+01 | 1.1532E+01 | 1.1340E+01 | 1.2164E+01 |
|  |  | STD | **1.4622E+00** | 1.8733E+00 | 2.1697E+00 | 2.8012E+00 | 2.2964E+00 | 2.5635E+00 | 2.3846E+00 |
|  | 3 | AVG | 1.5117E+01 | 1.5580E+01 | **1.5576E+01** | 1.4246E+01 | 1.2578E+01 | 1.1829E+01 | 1.3237E+01 |
|  |  | STD | 1.8815E+00 | **1.2485E+00** | 1.8358E+00 | 2.5843E+00 | 2.3381E+00 | 2.8475E+00 | 2.1731E+00 |
|  | 4 | AVG | 1.6160E+01 | 1.6216E+01 | **1.6004E+01** | 1.5156E+01 | 1.3833E+01 | 1.4241E+01 | 1.4049E+01 |
|  |  | STD | 1.7812E+00 | 2.0613E+00 | **1.3815E+00** | 2.1242E+00 | 2.3581E+00 | 2.2708E+00 | 2.3749E+00 |

**Table 3**. Comparison of mean and SD of the SSIM for all the algorithms segmentation of maize leaf spot images color images 1–5.

| Image | Thresholds | Item | GCLPSO | CLPSO | SCADE | m_ SCA | SSA | SCA | SMA |
| --- | --- | --- | --- | --- | --- | --- | --- | --- | --- |
| color image 01 | 2 | AVG | **5.7798E-01** | 5.7368E-01 | 5.6365E-01 | 5.6768E-01 | 4.3739E-01 | 4.6563E-01 | 4.8332E-01 |
|  |  | STD | 1.1608E-02 | **5.1596E-02** | 3.7185E-02 | 4.9653E-02 | 1.8215E-01 | 1.5947E-01 | 1.8934E-01 |
|  | 3 | AVG | **6.4999E-01** | 6.2909E-01 | 6.3468E-01 | 6.0259E-01 | 4.9621E-01 | 5.2883E-01 | 5.6104E-01 |
|  |  | STD | 4.7462E-02 | 5.8368E-02 | 7.1965E-02 | 1.2302E-01 | 1.9735E-01 | **6.8054E-02** | 7.5211E-02 |
|  | 4 | AVG | 6.5211E-01 | 6.4390E-01 | 6.3461E-01 | 6.5225E-01 | 5.7950E-01 | 5.3411E-01 | 6.1649E-01 |
|  |  | STD | 6.0197E-02 | 7.0716E-02 | 8.7516E-02 | 8.1893E-02 | 1.0432E-01 | **1.0068E-01** | 7.8360E-02 |
| color image 02 | 2 | AVG | **5.0026E-01** | 4.7642E-01 | 4.7265E-01 | 4.3912E-01 | 4.2923E-01 | 4.4213E-01 | 4.7128E-01 |
|  |  | STD | 8.5408E-02 | 1.0391E-01 | 9.7399E-02 | **1.2148E-01** | 1.3130E-01 | 1.0196E-01 | 1.0662E-01 |
|  | 3 | AVG | **5.8979E-01** | 5.5434E-01 | 5.0142E-01 | 5.5372E-01 | 5.3041E-01 | 4.4108E-01 | 4.7589E-01 |
|  |  | STD | 3.8509E-02 | 9.3858E-02 | 9.6890E-02 | 6.3649E-02 | 9.6239E-02 | 1.2543E-01 | **1.0829E-01** |
|  | 4 | AVG | 6.1120E-01 | 5.6835E-01 | 5.2988E-01 | 5.2676E-01 | 4.6856E-01 | 4.6021E-01 | 5.2947E-01 |
|  |  | STD | **4.4405E-02** | 1.0107E-01 | 1.1101E-01 | 1.2530E-01 | 1.3116E-01 | 1.3421E-01 | 1.2776E-01 |
| color image 03 | 2 | AVG | **6.0179E-01** | 5.9255E-01 | 5.9057E-01 | 5.5574E-01 | 4.7656E-01 | 5.4431E-01 | 4.9419E-01 |
|  |  | STD | **1.8332E-02** | 1.9932E-02 | 2.7814E-02 | 1.0154E-01 | 1.9657E-01 | 2.8908E-02 | 1.8074E-01 |
|  | 3 | AVG | **6.0569E-01** | 5.9531E-01 | 5.9630E-01 | 5.8926E-01 | 5.5432E-01 | 5.6079E-01 | 5.6707E-01 |
|  |  | STD | 3.7794E-02 | **4.9427E-02** | 5.6318E-02 | 3.4995E-02 | 1.3601E-01 | 3.9422E-02 | 1.0438E-01 |
|  | 4 | AVG | **6.2196E-01** | 6.0424E-01 | 6.1742E-01 | 5.9065E-01 | 5.4000E-01 | 5.5130E-01 | 6.0914E-01 |
|  |  | STD | 8.0604E-02 | 1.0996E-01 | 4.9307E-02 | 1.0592E-01 | 1.6353E-01 | 1.1580E-01 | 3.3514E-02 |
| color image 04 | 2 | AVG | 4.7747E-01 | **4.8042E-01** | 4.3991E-01 | 3.9258E-01 | 3.4523E-01 | 2.3237E-01 | 2.8131E-01 |
|  |  | STD | 2.3339E-02 | **1.7696E-02** | 1.3149E-01 | 1.5454E-01 | 1.6281E-01 | 1.6880E-01 | 1.6059E-01 |
|  | 3 | AVG | 5.9283E-01 | 5.4964E-01 | 5.6567E-01 | 4.9506E-01 | 3.8524E-01 | 4.6806E-01 | 4.4193E-01 |
|  |  | STD | 9.3048E-02 | 9.9416E-02 | 1.2122E-01 | 1.3210E-01 | 1.6441E-01 | **1.1503E-01** | 1.5032E-01 |
|  | 4 | AVG | 5.9749E-01 | 5.4425E-01 | 5.9368E-01 | 6.0014E-01 | 5.4674E-01 | 4.7862E-01 | 5.6110E-01 |
|  |  | STD | 1.2246E-01 | 9.5734E-02 | 1.0227E-01 | 1.0762E-01 | 1.0291E-01 | **9.0888E-02** | 1.0932E-01 |
| color image 05 | 2 | AVG | **4.0599E-01** | 3.8146E-01 | 3.8160E-01 | 2.8444E-01 | 2.5310E-01 | 2.3228E-01 | 2.8416E-01 |
|  |  | STD | **6.0735E-02** | 8.7795E-02 | 1.0748E-01 | 1.4699E-01 | 1.3250E-01 | 1.4108E-01 | 1.2959E-01 |
|  | 3 | AVG | 4.2899E-01 | 4.4976E-01 | **4.5709E-01** | 3.7838E-01 | 3.1503E-01 | 2.5957E-01 | 3.3994E-01 |
|  |  | STD | 9.0706E-02 | **5.6872E-02** | 9.4900E-02 | 1.2763E-01 | 1.2926E-01 | 1.4894E-01 | 1.0793E-01 |
|  | 4 | AVG | 4.8445E-01 | 4.8113E-01 | **4.8835E-01** | 4.2523E-01 | 3.6945E-01 | 3.8849E-01 | 3.7252E-01 |
|  |  | STD | 7.6006E-02 | 8.7002E-02 | **5.9096E-02** | 1.0459E-01 | 1.1548E-01 | 1.0872E-01 | 1.1559E-01 |

**Table 4**. Comparison of mean and SD of the FSIM of all the algorithms for segmentation of maize gray spot image color images 6–10.

| Image | Thresholds | Item | GCLPSO | CLPSO | SCADE | m_ SCA | SSA | SCA | SMA |
| --- | --- | --- | --- | --- | --- | --- | --- | --- | --- |
| color image 06 | 2 | AVG | **6.0338E-01** | 6.0005E-01 | 5.6926E-01 | 5.4913E-01 | 5.5468E-01 | 4.7857E-01 | 5.0953E-01 |
|  |  | STD | 3.2892E-02 | **4.1530E-02** | 5.6723E-02 | 7.6262E-02 | 8.0546E-02 | 6.5481E-02 | 8.0145E-02 |
|  | 3 | AVG | **6.3640E-01** | 6.3157E-01 | 6.3644E-01 | 6.0116E-01 | 5.8543E-01 | 5.5730E-01 | 5.9745E-01 |
|  |  | STD | 2.7497E-02 | 5.0463E-02 | 3.4673E-02 | 5.9452E-02 | 7.3481E-02 | **8.0389E-02** | 6.0917E-02 |
|  | 4 | AVG | 6.5546E-01 | 6.4788E-01 | 6.2494E-01 | 6.2912E-01 | 5.9569E-01 | 5.9350E-01 | 6.3407E-01 |
|  |  | STD | 3.9617E-02 | 4.4923E-02 | 3.9864E-02 | 5.1473E-02 | 6.4776E-02 | **6.6334E-02** | 3.0843E-02 |
| color image 07 | 2 | AVG | **5.3771E-01** | 5.3671E-01 | 5.3271E-01 | 5.3381E-01 | 5.0785E-01 | 4.7979E-01 | 4.8232E-01 |
|  |  | STD | 1.6655E-02 | 2.0228E-02 | 3.4277E-02 | **7.3905E-03** | 5.2560E-02 | 6.0021E-02 | 6.7386E-02 |
|  | 3 | AVG | **6.4016E-01** | 6.1023E-01 | 6.2193E-01 | 6.1465E-01 | 5.3114E-01 | 5.3661E-01 | 5.4555E-01 |
|  |  | STD | 3.1334E-02 | 4.9369E-02 | 5.1936E-02 | 6.8153E-02 | 8.1332E-02 | 2.9880E-02 | **5.2968E-02** |
|  | 4 | AVG | 6.5776E-01 | 6.2830E-01 | 6.4405E-01 | 6.3555E-01 | 6.0629E-01 | 5.5658E-01 | 6.1252E-01 |
|  |  | STD | **3.6196E-02** | 4.9547E-02 | 4.0362E-02 | 6.9339E-02 | 6.5522E-02 | 5.8306E-02 | 7.1674E-02 |
| color image 08 | 2 | AVG | **5.3633E-01** | 5.3725E-01 | 5.2809E-01 | 5.2200E-01 | 5.2323E-01 | 4.9248E-01 | 5.0828E-01 |
|  |  | STD | **1.8704E-02** | 2.1573E-02 | 2.2527E-02 | 2.6574E-02 | 3.0067E-02 | 1.5620E-02 | 2.6773E-02 |
|  | 3 | AVG | **5.8118E-01** | 5.7574E-01 | 5.6132E-01 | 5.3587E-01 | 5.1905E-01 | 5.3375E-01 | 5.3755E-01 |
|  |  | STD | 2.8260E-02 | **3.4858E-02** | 3.3368E-02 | 4.2627E-02 | 3.8914E-02 | 3.3349E-02 | 2.9101E-02 |
|  | 4 | AVG | **5.7836E-01** | 5.8670E-01 | 5.7998E-01 | 5.7061E-01 | 5.5101E-01 | 5.3751E-01 | 5.7132E-01 |
|  |  | STD | 3.8912E-02 | 3.6696E-02 | 4.0172E-02 | 4.5091E-02 | 4.0410E-02 | 3.4867E-02 | 3.7223E-02 |
| color image 09 | 2 | AVG | 5.9575E-01 | **5.7618E-01** | 5.9976E-01 | 5.6700E-01 | 5.6282E-01 | 5.1747E-01 | 5.2007E-01 |
|  |  | STD | 2.1776E-02 | **3.5296E-02** | 2.1592E-02 | 4.5699E-02 | 4.4755E-02 | 2.9775E-02 | 2.8657E-02 |
|  | 3 | AVG | 6.0594E-01 | 6.1584E-01 | 6.1621E-01 | 5.8707E-01 | 5.5860E-01 | 5.5714E-01 | 5.7267E-01 |
|  |  | STD | 4.3094E-02 | 3.0114E-02 | 4.1811E-02 | 4.6118E-02 | 5.0394E-02 | **4.0531E-02** | 4.3077E-02 |
|  | 4 | AVG | 6.3218E-01 | 6.1724E-01 | 6.3851E-01 | 6.1452E-01 | 5.7916E-01 | 5.7965E-01 | 5.8829E-01 |
|  |  | STD | 4.6813E-02 | 3.8128E-02 | 5.1853E-02 | 3.5671E-02 | 5.1762E-02 | **4.1404E-02** | 4.8563E-02 |
| color image 10 | 2 | AVG | **6.2135E-01** | 6.1848E-01 | 5.9521E-01 | 5.7366E-01 | 5.5704E-01 | 5.3128E-01 | 5.3678E-01 |
|  |  | STD | **1.4579E-02** | 1.2657E-02 | 5.1573E-02 | 5.4123E-02 | 4.5647E-02 | 4.6252E-02 | 3.6883E-02 |
|  | 3 | AVG | 6.3851E-01 | 6.3778E-01 | **6.6553E-01** | 6.1808E-01 | 5.6241E-01 | 5.8241E-01 | 5.9718E-01 |
|  |  | STD | 4.9286E-02 | **3.8648E-02** | 4.9265E-02 | 5.2550E-02 | 5.0826E-02 | 5.3273E-02 | 6.3420E-02 |
|  | 4 | AVG | 6.6256E-01 | 6.5387E-01 | **6.6681E-01** | 6.3520E-01 | 6.1280E-01 | 6.0197E-01 | 6.6246E-01 |
|  |  | STD | 6.2251E-02 | 5.0771E-02 | **5.0502E-02** | 5.2191E-02 | 5.2102E-02 | 5.2369E-02 | 5.0434E-02 |

**Table 5**. Comparison of mean and SD of the PSNR for all the algorithms segmentation of maize gray spot images color images 6–10.

| Image | Thresholds | Item | GCLPSO | CLPSO | SCADE | m_ SCA | SSA | SCA | SMA |
| --- | --- | --- | --- | --- | --- | --- | --- | --- | --- |
| color image 06 | 2 | AVG | **1.3342E+01** | 1.3298E+01 | 1.2132E+01 | 1.1703E+01 | 1.2004E+01 | 9.7428E+00 | 1.0863E+01 |
|  |  | STD | 1.4494E+00 | **1.7670E+00** | 1.9047E+00 | 2.3885E+00 | 2.6473E+00 | 2.2268E+00 | 2.6549E+00 |
|  | 3 | AVG | **1.4810E+01** | 1.4440E+01 | 1.4710E+01 | 1.3357E+01 | 1.3091E+01 | 1.2296E+01 | 1.3396E+01 |
|  |  | STD | 1.0647E+00 | 1.9970E+00 | 1.4500E+00 | 2.1213E+00 | 2.4164E+00 | **2.7663E+00** | 2.1332E+00 |
|  | 4 | AVG | 1.5478E+01 | 1.5217E+01 | 1.4407E+01 | 1.4541E+01 | 1.3304E+01 | 1.3638E+01 | 1.4655E+01 |
|  |  | STD | 1.6789E+00 | 1.6358E+00 | 1.3341E+00 | 1.7620E+00 | 2.1423E+00 | **2.2898E+00** | 1.4650E+00 |
| color image 07 | 2 | AVG | **1.4803E+01** | 1.4461E+01 | 1.4803E+01 | 1.4783E+01 | 1.3537E+01 | 1.2588E+01 | 1.2688E+01 |
|  |  | STD | 7.7412E-01 | 8.9621E-01 | 1.0111E+00 | **3.7884E-01** | 1.9463E+00 | 2.3267E+00 | 1.7979E+00 |
|  | 3 | AVG | **1.7664E+01** | 1.6748E+01 | 1.6851E+01 | 1.6986E+01 | 1.4294E+01 | 1.4602E+01 | 1.4755E+01 |
|  |  | STD | 1.0029E+00 | 1.4950E+00 | 1.5573E+00 | 2.0305E+00 | 2.5109E+00 | 1.4311E+00 | **1.6070E+00** |
|  | 4 | AVG | 1.8244E+01 | 1.7468E+01 | 1.7838E+01 | 1.7514E+01 | 1.6463E+01 | 1.5198E+01 | 1.6677E+01 |
|  |  | STD | **1.1208E+00** | 1.9463E+00 | 1.1383E+00 | 2.1896E+00 | 2.0391E+00 | 1.8911E+00 | 2.0467E+00 |
| color image 08 | 2 | AVG | **1.2750E+01** | 1.2448E+01 | 1.2276E+01 | 1.2010E+01 | 1.1742E+01 | 1.0751E+01 | 1.0938E+01 |
|  |  | STD | **7.6220E-01** | 1.3456E+00 | 9.0181E-01 | 1.5923E+00 | 2.7702E+00 | 2.2501E+00 | 2.6020E+00 |
|  | 3 | AVG | **1.3340E+01** | 1.3837E+01 | 1.2461E+01 | 1.2505E+01 | 1.1205E+01 | 1.2489E+01 | 1.2274E+01 |
|  |  | STD | 1.3820E+00 | **1.8295E+00** | 9.9670E-01 | 1.3435E+00 | 2.6988E+00 | 1.7464E+00 | 2.0595E+00 |
|  | 4 | AVG | **1.3371E+01** | 1.4444E+01 | 1.2689E+01 | 1.3122E+01 | 1.2544E+01 | 1.2140E+01 | 1.3031E+01 |
|  |  | STD | 1.9414E+00 | 1.6157E+00 | 1.7811E+00 | 1.7539E+00 | 2.6524E+00 | 2.7428E+00 | 1.7183E+00 |
| color image 09 | 2 | AVG | 1.3718E+01 | **1.3675E+01** | 1.3528E+01 | 1.2851E+01 | 1.2870E+01 | 1.0754E+01 | 1.1865E+01 |
|  |  | STD | 8.8644E-01 | **9.9383E-01** | 9.2599E-01 | 1.9085E+00 | 1.8515E+00 | 1.9522E+00 | 2.1542E+00 |
|  | 3 | AVG | 1.3520E+01 | 1.4272E+01 | 1.3891E+01 | 1.3646E+01 | 1.2793E+01 | 1.2857E+01 | 1.3605E+01 |
|  |  | STD | 1.0098E+00 | 9.7318E-01 | 8.7659E-01 | 1.4209E+00 | 2.0239E+00 | **1.8124E+00** | 1.0843E+00 |
|  | 4 | AVG | 1.4444E+01 | 1.4988E+01 | 1.4582E+01 | 1.4482E+01 | 1.3269E+01 | 1.4095E+01 | 1.3449E+01 |
|  |  | STD | 1.5524E+00 | 1.4034E+00 | 1.8577E+00 | 1.0239E+00 | 1.8220E+00 | **2.2875E+00** | 1.3753E+00 |
| color image 10 | 2 | AVG | **1.7320E+01** | 1.7335E+01 | 1.6808E+01 | 1.5542E+01 | 1.3046E+01 | 1.4480E+01 | 1.2187E+01 |
|  |  | STD | **7.8396E-01** | 4.9904E-01 | 1.0176E+00 | 2.8629E+00 | 3.8695E+00 | 2.7298E+00 | 3.5047E+00 |
|  | 3 | AVG | 1.7482E+01 | 1.7156E+01 | **1.7859E+01** | 1.6586E+01 | 1.4119E+01 | 1.5163E+01 | 1.6661E+01 |
|  |  | STD | 1.6446E+00 | **2.0562E+00** | 2.1397E+00 | 2.7698E+00 | 3.6622E+00 | 3.1538E+00 | 1.8450E+00 |
|  | 4 | AVG | 1.7722E+01 | 1.7670E+01 | **1.8254E+01** | 1.7406E+01 | 1.6710E+01 | 1.6184E+01 | 1.7998E+01 |
|  |  | STD | 2.9995E+00 | 2.5790E+00 | **1.4735E+00** | 1.8132E+00 | 2.2855E+00 | 2.6689E+00 | 8.7319E-01 |

**Table 6**. Comparison of mean and SD of the SSIM for all the algorithms segmentation of maize gray spot image color images 6–10.

| Image | Thresholds | Item | GCLPSO | CLPSO | SCADE | m_ SCA | SSA | SCA | SMA |
| --- | --- | --- | --- | --- | --- | --- | --- | --- | --- |
| color image 06 | 2 | AVG | **4.4008E-01** | 4.3355E-01 | 3.7262E-01 | 3.4383E-01 | 3.5645E-01 | 2.2077E-01 | 2.8677E-01 |
|  |  | STD | 7.2889E-02 | **8.7702E-02** | 1.0687E-01 | 1.3812E-01 | 1.4898E-01 | 1.2214E-01 | 1.5015E-01 |
|  | 3 | AVG | **5.1789E-01** | 4.9075E-01 | 5.1454E-01 | 4.3982E-01 | 4.1874E-01 | 3.6684E-01 | 4.3778E-01 |
|  |  | STD | 5.5198E-02 | 1.0207E-01 | 7.3913E-02 | 1.1668E-01 | 1.3395E-01 | **1.4901E-01** | 1.1585E-01 |
|  | 4 | AVG | 5.4754E-01 | 5.3412E-01 | 5.0578E-01 | 5.0302E-01 | 4.3620E-01 | 4.3795E-01 | 5.0814E-01 |
|  |  | STD | 8.1275E-02 | 8.5877E-02 | 7.7131E-02 | 9.4469E-02 | 1.1900E-01 | **1.1873E-01** | 7.3352E-02 |
| color image 07 | 2 | AVG | **3.9307E-01** | 3.8074E-01 | 3.9453E-01 | 3.9419E-01 | 3.2398E-01 | 2.7110E-01 | 2.8783E-01 |
|  |  | STD | 3.0862E-02 | 3.8847E-02 | 3.9045E-02 | **1.6907E-02** | 1.0731E-01 | 1.2614E-01 | 9.9108E-02 |
|  | 3 | AVG | **5.3573E-01** | 4.8551E-01 | 4.9818E-01 | 5.0076E-01 | 3.6569E-01 | 3.8566E-01 | 3.9127E-01 |
|  |  | STD | 4.9623E-02 | 7.2799E-02 | 8.7254E-02 | 1.0336E-01 | 1.3054E-01 | 6.7483E-02 | **8.0112E-02** |
|  | 4 | AVG | 5.6770E-01 | 5.2342E-01 | 5.5270E-01 | 5.2857E-01 | 4.7804E-01 | 4.1555E-01 | 4.9051E-01 |
|  |  | STD | **5.0304E-02** | 9.7628E-02 | 5.7620E-02 | 1.1331E-01 | 1.0271E-01 | 9.4312E-02 | 1.0477E-01 |
| color image 08 | 2 | AVG | **4.0672E-01** | 3.9569E-01 | 3.8361E-01 | 3.6986E-01 | 3.5137E-01 | 2.9056E-01 | 3.1000E-01 |
|  |  | STD | **3.6500E-02** | 5.5863E-02 | 4.4215E-02 | 8.8014E-02 | 1.4822E-01 | 1.2986E-01 | 1.5454E-01 |
|  | 3 | AVG | **4.4439E-01** | 4.5784E-01 | 4.0582E-01 | 3.9892E-01 | 3.4080E-01 | 3.8864E-01 | 3.9054E-01 |
|  |  | STD | 5.6615E-02 | **7.2714E-02** | 4.9851E-02 | 6.6176E-02 | 1.3263E-01 | 9.1350E-02 | 1.1061E-01 |
|  | 4 | AVG | **4.4837E-01** | 4.8743E-01 | 4.2744E-01 | 4.3568E-01 | 4.0215E-01 | 3.7033E-01 | 4.4072E-01 |
|  |  | STD | 8.2988E-02 | 5.9896E-02 | 8.5245E-02 | 8.1053E-02 | 1.2638E-01 | 1.4310E-01 | 7.4571E-02 |
| color image 09 | 2 | AVG | 3.1802E-01 | **3.0810E-01** | 3.1146E-01 | 2.6340E-01 | 2.6222E-01 | 1.3432E-01 | 1.9315E-01 |
|  |  | STD | 4.8899E-02 | **5.8815E-02** | 5.4649E-02 | 1.1300E-01 | 1.0926E-01 | 1.0752E-01 | 1.1843E-01 |
|  | 3 | AVG | 3.1519E-01 | 3.6246E-01 | 3.4210E-01 | 3.1658E-01 | 2.6123E-01 | 2.6473E-01 | 3.0478E-01 |
|  |  | STD | 6.4019E-02 | 5.3698E-02 | 5.3817E-02 | 8.5703E-02 | 1.1999E-01 | **9.6129E-02** | 6.7402E-02 |
|  | 4 | AVG | 3.8342E-01 | 3.9993E-01 | 3.8841E-01 | 3.7303E-01 | 2.9485E-01 | 3.3623E-01 | 3.0552E-01 |
|  |  | STD | 9.5533E-02 | 7.2476E-02 | 1.0628E-01 | 5.9193E-02 | 1.0803E-01 | **1.2354E-01** | 8.7093E-02 |
| color image 10 | 2 | AVG | **5.4589E-01** | 5.4622E-01 | 5.2102E-01 | 4.5208E-01 | 3.2895E-01 | 4.0723E-01 | 2.8704E-01 |
|  |  | STD | **3.8476E-02** | 2.7921E-02 | 5.1283E-02 | 1.4094E-01 | 1.9086E-01 | 1.4032E-01 | 1.7343E-01 |
|  | 3 | AVG | 5.6197E-01 | 5.4922E-01 | **5.8906E-01** | 5.0719E-01 | 3.8203E-01 | 4.5012E-01 | 5.1018E-01 |
|  |  | STD | 7.6465E-02 | **8.8873E-02** | 1.0429E-01 | 1.4042E-01 | 1.8066E-01 | 1.5495E-01 | 9.4499E-02 |
|  | 4 | AVG | 5.7398E-01 | 5.7409E-01 | **6.1131E-01** | 5.5033E-01 | 5.1325E-01 | 5.0511E-01 | 5.8875E-01 |
|  |  | STD | 1.2971E-01 | 1.1279E-01 | **6.7079E-02** | 9.6966E-02 | 1.1479E-01 | 1.3145E-01 | 6.0023E-02 |

**Table 7**. Comparison of mean and SD of the FSIM of all the algorithms for segmentation of maize rust images color images 11–15.

| Image | Thresholds | Item | GCLPSO | CLPSO | SCADE | m_ SCA | SSA | SCA | SMA |
| --- | --- | --- | --- | --- | --- | --- | --- | --- | --- |
| color image 11 | 2 | AVG | **5.6707E-01** | 5.9453E-01 | 5.7798E-01 | 5.6467E-01 | 5.8159E-01 | 5.3685E-01 | 5.7670E-01 |
|  |  | STD | 2.8071E-02 | **4.3471E-02** | 4.2184E-02 | 2.6985E-02 | 3.8179E-02 | 7.2511E-02 | 5.7474E-02 |
|  | 3 | AVG | **6.9378E-01** | 6.8681E-01 | 5.9110E-01 | 6.3601E-01 | 6.1832E-01 | 6.1249E-01 | 6.2770E-01 |
|  |  | STD | 5.6150E-02 | 7.7096E-02 | 7.2108E-02 | 6.8232E-02 | 7.3373E-02 | **6.3924E-02** | 5.8882E-02 |
|  | 4 | AVG | 7.0607E-01 | 6.8522E-01 | 6.3735E-01 | 6.8239E-01 | 6.8383E-01 | 6.2759E-01 | 6.4412E-01 |
|  |  | STD | 7.1008E-02 | 7.0647E-02 | 9.1422E-02 | 6.6256E-02 | 7.1313E-02 | **7.9184E-02** | 6.4725E-02 |
| color image 12 | 2 | AVG | **6.4910E-01** | 6.4509E-01 | 6.3539E-01 | 6.4078E-01 | 6.1721E-01 | 5.9342E-01 | 6.1031E-01 |
|  |  | STD | 2.2045E-02 | 2.1002E-02 | 2.2472E-02 | **2.3702E-02** | 4.3346E-02 | 4.3484E-02 | 4.6752E-02 |
|  | 3 | AVG | **7.0612E-01** | 6.7339E-01 | 6.7018E-01 | 7.0478E-01 | 6.6505E-01 | 6.4770E-01 | 6.5900E-01 |
|  |  | STD | 2.7120E-02 | 3.5762E-02 | 4.4499E-02 | 3.9076E-02 | 4.6602E-02 | 3.4285E-02 | **3.9906E-02** |
|  | 4 | AVG | 7.1854E-01 | 7.0764E-01 | 7.0041E-01 | 7.1953E-01 | 6.7655E-01 | 6.9676E-01 | 6.9172E-01 |
|  |  | STD | **3.1150E-02** | 3.1996E-02 | 4.2172E-02 | 2.6493E-02 | 4.7422E-02 | 2.8103E-02 | 4.7203E-02 |
| color image 13 | 2 | AVG | **5.7003E-01** | 5.5026E-01 | 5.7043E-01 | 5.6337E-01 | 5.5577E-01 | 4.9516E-01 | 5.1571E-01 |
|  |  | STD | **4.3458E-02** | 4.1402E-02 | 6.1496E-02 | 2.5034E-02 | 4.4979E-02 | 8.2346E-02 | 6.0922E-02 |
|  | 3 | AVG | **7.0961E-01** | 6.9566E-01 | 6.4008E-01 | 6.4123E-01 | 5.9860E-01 | 5.8284E-01 | 5.6501E-01 |
|  |  | STD | 6.1831E-02 | **7.2431E-02** | 9.3125E-02 | 7.0119E-02 | 8.0153E-02 | 6.7887E-02 | 6.1100E-02 |
|  | 4 | AVG | **7.2769E-01** | 7.0051E-01 | 6.8771E-01 | 6.7387E-01 | 6.1062E-01 | 6.3244E-01 | 6.3960E-01 |
|  |  | STD | 8.0055E-02 | 7.8663E-02 | 9.2990E-02 | 8.3675E-02 | 8.5069E-02 | 7.9620E-02 | 7.0286E-02 |
| color image 14 | 2 | AVG | 6.2345E-01 | **6.1016E-01** | 6.4872E-01 | 6.1488E-01 | 6.0217E-01 | 5.8903E-01 | 5.6091E-01 |
|  |  | STD | 3.8838E-02 | **3.8092E-02** | 5.1599E-02 | 3.1163E-02 | 6.6478E-02 | 6.7041E-02 | 5.5032E-02 |
|  | 3 | AVG | 7.2283E-01 | 7.1871E-01 | 7.1708E-01 | 7.0108E-01 | 6.4253E-01 | 6.6582E-01 | 6.5490E-01 |
|  |  | STD | 6.0148E-02 | 6.3584E-02 | 5.4035E-02 | 7.1498E-02 | 7.7352E-02 | **6.2482E-02** | 7.6894E-02 |
|  | 4 | AVG | 7.5703E-01 | 7.4315E-01 | 7.5085E-01 | 7.5808E-01 | 7.1327E-01 | 6.8798E-01 | 7.2644E-01 |
|  |  | STD | 4.7923E-02 | 5.2580E-02 | 5.2867E-02 | 5.0708E-02 | 8.2370E-02 | **5.9289E-02** | 6.4412E-02 |
| color image 15 | 2 | AVG | **7.0194E-01** | 7.0526E-01 | 6.7845E-01 | 6.8394E-01 | 6.8504E-01 | 6.7012E-01 | 6.6436E-01 |
|  |  | STD | **2.7236E-02** | 2.5492E-02 | 2.1654E-02 | 2.1336E-02 | 2.4228E-02 | 3.5126E-02 | 5.1167E-02 |
|  | 3 | AVG | 7.5967E-01 | 7.4891E-01 | **7.2759E-01** | 7.3602E-01 | 7.0632E-01 | 6.9809E-01 | 7.1360E-01 |
|  |  | STD | 1.6717E-02 | **2.4624E-02** | 4.2145E-02 | 3.9411E-02 | 5.4503E-02 | 3.3817E-02 | 4.6796E-02 |
|  | 4 | AVG | 7.8847E-01 | 7.5114E-01 | **7.4301E-01** | 7.7001E-01 | 7.5510E-01 | 7.3733E-01 | 7.5837E-01 |
|  |  | STD | 3.4097E-02 | 3.9084E-02 | **3.0573E-02** | 2.6927E-02 | 4.3002E-02 | 3.0054E-02 | 4.7377E-02 |

**Table 8**. Comparison of mean and SD of the PSNR for all the algorithms segmentation of maize rust images color images 11–15.

| Image | Thresholds | Item | GCLPSO | CLPSO | SCADE | m_ SCA | SSA | SCA | SMA |
| --- | --- | --- | --- | --- | --- | --- | --- | --- | --- |
| color image 11 | 2 | AVG | **1.4307E+01** | 1.5314E+01 | 1.4728E+01 | 1.4247E+01 | 1.4934E+01 | 1.2618E+01 | 1.4492E+01 |
|  |  | STD | 1.2647E+00 | **1.4315E+00** | 1.6566E+00 | 1.1495E+00 | 1.3222E+00 | 3.2126E+00 | 1.9718E+00 |
|  | 3 | AVG | **1.7483E+01** | 1.7415E+01 | 1.4757E+01 | 1.5886E+01 | 1.5766E+01 | 1.5595E+01 | 1.5790E+01 |
|  |  | STD | 2.1618E+00 | 2.5500E+00 | 2.3970E+00 | 1.7006E+00 | 2.1008E+00 | **2.0238E+00** | 1.5332E+00 |
|  | 4 | AVG | 1.8008E+01 | 1.7057E+01 | 1.5944E+01 | 1.7283E+01 | 1.7651E+01 | 1.5856E+01 | 1.6185E+01 |
|  |  | STD | 2.6678E+00 | 2.2647E+00 | 3.2263E+00 | 2.1998E+00 | 2.2137E+00 | **2.8584E+00** | 1.6954E+00 |
| color image 12 | 2 | AVG | **1.6187E+01** | 1.5868E+01 | 1.6247E+01 | 1.6062E+01 | 1.5427E+01 | 1.4296E+01 | 1.4783E+01 |
|  |  | STD | 9.2644E-01 | 1.0724E+00 | 1.1783E+00 | **5.5537E-01** | 2.0352E+00 | 2.4066E+00 | 2.0420E+00 |
|  | 3 | AVG | **1.9314E+01** | 1.8168E+01 | 1.7690E+01 | 1.8989E+01 | 1.7683E+01 | 1.6698E+01 | 1.6884E+01 |
|  |  | STD | 1.2073E+00 | 1.6797E+00 | 1.9765E+00 | 1.7149E+00 | 2.2707E+00 | 1.4980E+00 | **1.5113E+00** |
|  | 4 | AVG | 1.9934E+01 | 1.9442E+01 | 1.9379E+01 | 1.9457E+01 | 1.8019E+01 | 1.8955E+01 | 1.8390E+01 |
|  |  | STD | **1.2715E+00** | 1.5518E+00 | 1.5589E+00 | 1.1295E+00 | 2.2304E+00 | 1.0681E+00 | 1.8837E+00 |
| color image 13 | 2 | AVG | **1.4033E+01** | 1.3562E+01 | 1.4543E+01 | 1.4436E+01 | 1.3945E+01 | 1.1439E+01 | 1.2525E+01 |
|  |  | STD | **2.1063E+00** | 2.3562E+00 | 2.5748E+00 | 1.4459E+00 | 2.1197E+00 | 3.4078E+00 | 2.7290E+00 |
|  | 3 | AVG | **1.8440E+01** | 1.8137E+01 | 1.6239E+01 | 1.6398E+01 | 1.5074E+01 | 1.4796E+01 | 1.4260E+01 |
|  |  | STD | 1.8921E+00 | **2.2855E+00** | 3.0566E+00 | 2.2982E+00 | 3.0411E+00 | 2.5254E+00 | 2.2225E+00 |
|  | 4 | AVG | **1.9003E+01** | 1.8087E+01 | 1.8384E+01 | 1.7624E+01 | 1.5404E+01 | 1.6230E+01 | 1.6507E+01 |
|  |  | STD | 2.9273E+00 | 2.7184E+00 | 3.1004E+00 | 2.6563E+00 | 3.0725E+00 | 2.3761E+00 | 2.1016E+00 |
| color image 14 | 2 | AVG | 1.4906E+01 | **1.4514E+01** | 1.6161E+01 | 1.4600E+01 | 1.4226E+01 | 1.4161E+01 | 1.2879E+01 |
|  |  | STD | 1.7311E+00 | **1.7921E+00** | 1.9648E+00 | 1.6587E+00 | 2.5452E+00 | 2.1157E+00 | 1.7820E+00 |
|  | 3 | AVG | 1.8854E+01 | 1.8693E+01 | 1.8659E+01 | 1.7945E+01 | 1.6097E+01 | 1.6773E+01 | 1.6182E+01 |
|  |  | STD | 2.3175E+00 | 2.4914E+00 | 2.0686E+00 | 3.0034E+00 | 2.8425E+00 | **2.2900E+00** | 3.0623E+00 |
|  | 4 | AVG | 2.0447E+01 | 1.9806E+01 | 2.0220E+01 | 2.0235E+01 | 1.8762E+01 | 1.7756E+01 | 1.9081E+01 |
|  |  | STD | 1.7742E+00 | 2.0313E+00 | 1.9588E+00 | 2.1361E+00 | 2.7686E+00 | **2.0951E+00** | 2.5396E+00 |
| color image 15 | 2 | AVG | **1.6504E+01** | 1.6371E+01 | 1.5422E+01 | 1.5578E+01 | 1.5729E+01 | 1.4367E+01 | 1.4398E+01 |
|  |  | STD | **8.6621E-01** | 7.4956E-01 | 1.5872E+00 | 8.7374E-01 | 8.8204E-01 | 2.2842E+00 | 2.3495E+00 |
|  | 3 | AVG | 2.0311E+01 | 2.0018E+01 | **1.8379E+01** | 1.8722E+01 | 1.7019E+01 | 1.6687E+01 | 1.7147E+01 |
|  |  | STD | 1.0504E+00 | **1.2425E+00** | 2.5732E+00 | 2.3320E+00 | 3.1577E+00 | 2.0417E+00 | 2.4314E+00 |
|  | 4 | AVG | 2.1855E+01 | 1.9802E+01 | **1.9381E+01** | 2.0827E+01 | 2.0210E+01 | 1.8961E+01 | 1.9944E+01 |
|  |  | STD | 1.8913E+00 | 2.3101E+00 | **1.8686E+00** | 1.5346E+00 | 2.3083E+00 | 1.8721E+00 | 2.6602E+00 |

**Table 9**. Comparison of mean and SD of the SSIM for all the algorithms segmentation of maize rust disease images color images 11–15.

| Image | Thresholds | Item | GCLPSO | CLPSO | SCADE | m_ SCA | SSA | SCA | SMA |
| --- | --- | --- | --- | --- | --- | --- | --- | --- | --- |
| color image 11 | 2 | AVG | **4.8601E-01** | 5.2568E-01 | 5.0216E-01 | 4.8616E-01 | 5.0992E-01 | 4.1229E-01 | 5.0028E-01 |
|  |  | STD | 3.6366E-02 | **5.5591E-02** | 5.7757E-02 | 3.7376E-02 | 4.9072E-02 | 1.3902E-01 | 8.3751E-02 |
|  | 3 | AVG | **6.1581E-01** | 6.1148E-01 | 5.0909E-01 | 5.5719E-01 | 5.4586E-01 | 5.4000E-01 | 5.5506E-01 |
|  |  | STD | 6.9016E-02 | 8.9782E-02 | 8.6862E-02 | 6.8840E-02 | 7.6192E-02 | **7.7374E-02** | 6.4097E-02 |
|  | 4 | AVG | 6.3090E-01 | 6.0522E-01 | 5.5332E-01 | 6.0750E-01 | 6.1534E-01 | 5.4540E-01 | 5.6940E-01 |
|  |  | STD | 8.7220E-02 | 8.0916E-02 | 1.1609E-01 | 7.4196E-02 | 7.8048E-02 | **1.0025E-01** | 6.8149E-02 |
| color image 12 | 2 | AVG | **5.9742E-01** | 5.8845E-01 | 5.9593E-01 | 5.9674E-01 | 5.8060E-01 | 5.3264E-01 | 5.5790E-01 |
|  |  | STD | 2.3570E-02 | 2.7400E-02 | 3.6055E-02 | **1.4671E-02** | 4.7339E-02 | 7.5269E-02 | 6.3862E-02 |
|  | 3 | AVG | **6.9942E-01** | 6.5880E-01 | 6.4578E-01 | 6.8758E-01 | 6.4726E-01 | 6.1620E-01 | 6.2769E-01 |
|  |  | STD | 3.7337E-02 | 4.6565E-02 | 6.1796E-02 | 5.3444E-02 | 6.4870E-02 | 4.9548E-02 | **4.5298E-02** |
|  | 4 | AVG | 7.1839E-01 | 7.0115E-01 | 6.9858E-01 | 7.0556E-01 | 6.5573E-01 | 6.8449E-01 | 6.7222E-01 |
|  |  | STD | **3.9894E-02** | 3.8801E-02 | 5.2835E-02 | 3.4448E-02 | 6.3084E-02 | 3.3150E-02 | 5.8833E-02 |
| color image 13 | 2 | AVG | **5.0338E-01** | 4.7931E-01 | 5.1729E-01 | 5.1925E-01 | 4.9766E-01 | 3.8366E-01 | 4.3636E-01 |
|  |  | STD | **8.2617E-02** | 9.8199E-02 | 1.0644E-01 | 5.7299E-02 | 8.9540E-02 | 1.4620E-01 | 1.2313E-01 |
|  | 3 | AVG | **6.6740E-01** | 6.5383E-01 | 5.8702E-01 | 5.9286E-01 | 5.3886E-01 | 5.3146E-01 | 5.1072E-01 |
|  |  | STD | 6.2881E-02 | **7.7799E-02** | 1.1388E-01 | 8.4793E-02 | 1.2033E-01 | 9.6584E-02 | 9.2565E-02 |
|  | 4 | AVG | **6.8387E-01** | 6.5410E-01 | 6.5514E-01 | 6.3249E-01 | 5.5151E-01 | 5.8859E-01 | 5.9642E-01 |
|  |  | STD | 9.3015E-02 | 8.6763E-02 | 1.0325E-01 | 9.0477E-02 | 1.2073E-01 | 8.3235E-02 | 7.7078E-02 |
| color image 14 | 2 | AVG | 5.6499E-01 | **5.4769E-01** | 6.0564E-01 | 5.5198E-01 | 5.3002E-01 | 5.2373E-01 | 4.7432E-01 |
|  |  | STD | 6.6232E-02 | **7.3002E-02** | 7.2295E-02 | 6.2752E-02 | 1.0113E-01 | 9.2163E-02 | 7.9725E-02 |
|  | 3 | AVG | 6.9613E-01 | 6.8984E-01 | 6.9126E-01 | 6.6375E-01 | 5.9431E-01 | 6.2840E-01 | 6.0334E-01 |
|  |  | STD | 7.7703E-02 | 8.2746E-02 | 6.7073E-02 | 1.0036E-01 | 1.0762E-01 | **8.0368E-02** | 1.0601E-01 |
|  | 4 | AVG | 7.4009E-01 | 7.2208E-01 | 7.3348E-01 | 7.3723E-01 | 6.8576E-01 | 6.5696E-01 | 6.9864E-01 |
|  |  | STD | 5.6215E-02 | 6.2188E-02 | 6.1066E-02 | 6.3921E-02 | 9.9528E-02 | **6.9825E-02** | 8.1733E-02 |
| color image 15 | 2 | AVG | **6.0653E-01** | 6.0518E-01 | 5.7563E-01 | 5.8437E-01 | 5.8935E-01 | 5.4200E-01 | 5.5692E-01 |
|  |  | STD | **2.1441E-02** | 1.8036E-02 | 4.6301E-02 | 2.6494E-02 | 2.6806E-02 | 6.9672E-02 | 5.8346E-02 |
|  | 3 | AVG | 6.9125E-01 | 6.8474E-01 | **6.4667E-01** | 6.5612E-01 | 6.1621E-01 | 6.0939E-01 | 6.2308E-01 |
|  |  | STD | 1.8614E-02 | **2.6369E-02** | 5.6208E-02 | 5.1810E-02 | 7.4840E-02 | 4.6402E-02 | 5.2723E-02 |
|  | 4 | AVG | 7.2167E-01 | 6.8123E-01 | **6.7028E-01** | 7.0041E-01 | 6.8715E-01 | 6.6231E-01 | 6.8441E-01 |
|  |  | STD | 3.9861E-02 | 5.0490E-02 | **3.9107E-02** | 3.2180E-02 | 4.6607E-02 | 3.9917E-02 | 5.4711E-02 |
